# Interactions between Growth of Muscle and Stature: Mechanisms Involved and Their Nutritional Sensitivity to Dietary Protein: The Protein-Stat Revisited

**DOI:** 10.3390/nu13030729

**Published:** 2021-02-25

**Authors:** D Joe Millward

**Affiliations:** Department of Nutritional Sciences, Faculty of Health and Medical Sciences, University of Surrey, Guildford GU2 7XH, UK; d.millward@surrey.ac.uk

**Keywords:** dietary-protein, growth, muscle, bone, satellite-cells, mechanotransduction, protein-synthesis, insulin, amino acids, IGF-1

## Abstract

Childhood growth and its sensitivity to dietary protein is reviewed within a Protein-Stat model of growth regulation. The coordination of growth of muscle and stature is a combination of genetic programming, and of two-way mechanical interactions involving the mechanotransduction of muscle growth through stretching by bone length growth, the core Protein-Stat feature, and the strengthening of bone through muscle contraction via the mechanostat. Thus, growth in bone length is the initiating event and this is always observed. Endocrine and cellular mechanisms of growth in stature are reviewed in terms of the growth hormone-insulin like growth factor-1 (GH-IGF-1) and thyroid axes and the sex hormones, which together mediate endochondral ossification in the growth plate and bone lengthening. Cellular mechanisms of muscle growth during development are then reviewed identifying (a) the difficulties posed by the need to maintain its ultrastructure during myofibre hypertrophy within the extracellular matrix and the concept of muscle as concentric “bags” allowing growth to be conceived as bag enlargement and filling, (b) the cellular and molecular mechanisms involved in the mechanotransduction of satellite and mesenchymal stromal cells, to enable both connective tissue remodelling and provision of new myonuclei to aid myofibre hypertrophy and (c) the implications of myofibre hypertrophy for protein turnover within the myonuclear domain. Experimental data from rodent and avian animal models illustrate likely changes in DNA domain size and protein turnover during developmental and stretch-induced muscle growth and between different muscle fibre types. Growth of muscle in male rats during adulthood suggests that “bag enlargement” is achieved mainly through the action of mesenchymal stromal cells. Current understanding of the nutritional regulation of protein deposition in muscle, deriving from experimental studies in animals and human adults, is reviewed, identifying regulation by amino acids, insulin and myofibre volume changes acting to increase both ribosomal capacity and efficiency of muscle protein synthesis via the mechanistic target of rapamycin complex 1 (mTORC1) and the phenomenon of a “bag-full” inhibitory signal has been identified in human skeletal muscle. The final section deals with the nutritional sensitivity of growth of muscle and stature to dietary protein in children. Growth in length/height as a function of dietary protein intake is described in the context of the breastfed child as the normative growth model, and the “Early Protein Hypothesis” linking high protein intakes in infancy to later adiposity. The extensive paediatric studies on serum IGF-1 and child growth are reviewed but their clinical relevance is of limited value for understanding growth regulation; a role in energy metabolism and homeostasis, acting with insulin to mediate adiposity, is probably more important. Information on the influence of dietary protein on muscle mass per se as opposed to lean body mass is limited but suggests that increased protein intake in children is unable to promote muscle growth in excess of that linked to genotypic growth in length/height. One possible exception is milk protein intake, which cohort and cross-cultural studies suggest can increase height and associated muscle growth, although such effects have yet to be demonstrated by randomised controlled trials.

## 1. Introduction

The outcome of the end of puberty is the young adult with a stature and muscularity determined by their genome, epigenetic modifications during foetal growth, and the extent to which their postnatal environment has provided adequate nutrition in a caring household with minimal stress from pathogens and social disruption. Stature and muscularity are both important outcomes for the individual and for the society in which they live. The secular trend in increasing population height is a clear demonstration of Tanner’s observation that growth is a “mirror of the conditions of society”, especially the “nutritional and hygienic status” of the population [1] and increases in population height are associated with decreased all-cause mortality and increased longevity [2]. For muscularity, it is clear that muscle’s essential motor function means that the regulation of its growth in children and maintenance in adult life, is key to understanding human health and wellbeing throughout the life cycle. Thus, low muscle mass and strength during childhood contribute to several adverse health outcomes [3], muscle mass and strength in adolescent men is inversely associated with later cardiovascular disease (CVD), events and CVD mortality in middle age [4], and low lean body mass in adult men predicts an increased risk of all-cause mortality and mortality from cardiovascular disease and cancer, explaining the obesity paradox of increasing risk associate with low body mass index, (BMI), [5]. Because muscle is a primary target organ for insulin action and glucose homeostasis then the relative size of the muscle mass is inversely related to insulin resistance [6], a major component of the mechanism linking muscle mass to mortality via its influence on the development of diabetes. This review is concerned with the mechanisms of the nutritional regulation of postnatal growth, especially that of skeletal muscle and its relationship with growth in stature, and the extent to which they are influenced by dietary protein. The mechanistic framework is that of the “Protein-Stat” a model for growth regulation described some years ago, based on an interaction between linear growth of bone and muscle growth [7].

In fact, there has been very little discussion of the Protein-Stat in the 25 years since it was published. The intention here is to revisit and update the details of the model relating to the coordination of growth of muscle and stature, reviewing the cellular and molecular mechanisms involved, how protein deposition in muscle is regulated at the level of protein synthesis and proteolysis, and how growth in stature and muscle in infants and children, especially in relation to feeding modalities and dietary protein intake, conforms to the Protein-Stat model. The other component of the model, the regulation of appetite by an aminostatic appetite mechanism [7], will not be discussed here.

## 2. Coordination of Growth of Muscle and Stature

### 2.1. Genetics and Programming

The growth potential of an individual in height and overall shape, mainly a function of bone growth, is genetically determined and each individual will follow a growth curve canalised in terms of both extent and time course if conditions are favourable [8], i.e., a diet which can exert an appropriate regulatory anabolic drive on growth [9] and provide necessary substrates, in an environment which presents minimal inflammatory challenges. While mechanical loading can influence bone density, length growth is relatively insensitive to physiological dynamic loading, although direct compressive stress on the growth plate inhibits growth [10].

In terms of the time course of postnatal growth, one widely quoted model [11] involves three additive and partly superimposed phases of postnatal linear-growth from birth to maturity, i.e., infancy, childhood and puberty (the ICP model). When interrupted by malnutrition or infection there is usually some period of catch-up growth [12], i.e., a self-correcting response returning the growth pattern to the individual growth channel, probably involving the delayed senescence of the growth plate.

The nature of the genetic programming is quite complex. Genetic factors are often estimated to account for 80% of the variation in height, the best-known polygenic trait. Genome-wide association studies of height in adults of recent European origin indicated >3000 single nucleotide polymorphism, (SNP), variants which explained 35% of the variation, clustered at >700 genomic loci of which >600 were genes and >700 were methylation sites [13]. Height-associated genes were significantly enriched among genes contributing to skeletal growth, cartilage and connective tissue development. The genetic programming of the time courses of linear-growth, especially in relation to events in the growth plate which mediate the slowing down of the initial very rapid foetal, early infancy growth phase with eventual cessation of linear growth after puberty, is particularly complex [14], as are the mechanisms which link linear growth to the growth of other organs and tissues. The progressive decline in cellular proliferation throughout the organism may result from a programmed downregulation of a large set of growth-promoting genes [14]. Height is also influenced by foetal programming as indicated by its relationship with birthweight, shown for example in early adolescent boys and girls [15].

As for the time course of muscle growth, there is much less information. The ultimate adult size of appendicular muscle mass is highly heritable, likely covariant-adjusted values were 68% and 94% in men and women of variable ages, respectively, in one study [16], and 42% for lower leg lean mass of elderly adults in Framingham, after adjustment [17]. Prenatal growth appears to program postnatal muscle growth with muscle mass a function of birth weight both in late adolescence, especially in boys [18] and in early adolescence in boys [15]. This phenomenon has been confirmed by Mendelian randomisation for subjects in the UK biobank cohort. Thus, SNPs predicting maternal effects on birth weight, independent of foetal genetics and excluding SNPs related to height, indicated that birth weight was positively associated with fat-free mass and grip strength in adults [18].

In children, skeletal muscle mass as a proportion of dual energy X-ray absorptiometry, (DEXA)-measured lean body mass is low in early life increasing with age, one study reporting that between the ages of 5 and 18 y, appendicular muscle mass, (about 79% of total muscle mass in adults [19]), accounts for only 33% of the total body fat-free mass, (FFM), in 5–7-year-olds rising to 42% at 18 y [20]. Thus, total muscle mass accounts for about 41% FFM (33/0.79) in school children increasing to about 53% at the end of puberty.

The bone-length–muscle-mass relationship may be modifiable by early life programming. Foetal muscle development, as indicated by muscle-mass and bodyweight at birth, is a determinant of grip strength throughout the life course [21] an influence also observed in 9-year-old Indian children [22].

### 2.2. Muscle and Bone Growth Interrelationships: The Protein-Stat and the Mechanostat

The regulation of muscle growth was described some years ago in the context of a Protein-Stat model for the control of growth, especially the protein content of the lean body mass which was under loose control, within which its major constituent, skeletal muscle protein, was tightly controlled [7]. The central feature of the model is an interaction between linear growth of bone, protein deposition in skeletal muscle and dietary protein intake. Several controllers are identified. The first is a dietary controller, which is the minimum protein concentration required to exert a sufficient anabolic drive or regulatory influence on linear bone growth to allow the genetic programming of its postnatal growth trajectory to be expressed, and to permit muscle myofibre growth. The “anabolic drive” of signalling is mediated by the endocrine system and amino acids subsequent to dietary protein intake, acting on bone length growth and enabling protein deposition in muscle. The second controller is internal, namely linear bone growth which mediates, through the mechanotransduction by passive stretch of muscle, myofibre growth capacity at the level of the architecture of the muscle extracellular matrix (ECM). This defines myofibre volume and potential protein content, with the growth of most other organs secondary to this interaction. The third controller is appetite control by an amino-static appetite mechanism activated by the metabolic demand for amino acids, which allows food protein intake to match capacity and demand.

The biomechanical relationship between muscle and bone growth [7,23,24,25,26] was a central feature of the model and if correct leads to the important prediction that growth in bone length should precede growth in muscle mass, a characteristic of bone and muscle growth which was not a mainstream view at the time or since. In fact, most discussion of this has been related to the bone-focused mechanostat [27] which describes how bones adapt their strength to the mechanical loads on them by muscle. Thus, the increase and subsequent decline in specific bone strength throughout the life cycle is viewed as a consequence of associated muscle action, so that increasing muscle mass and strength in the young and sarcopenia in the old are seen as the primary drivers of increases and later decreases in bone strength. The evidence for this includes, for example, cross-sectional and longitudinal studies of calf muscle size and tibial growth in young girls, which suggest that muscle size is an important determinant of bone strength development through increases in cortical and trabecular volumetric bone mineral density (BMD) [28]. In addition, in healthy prepubertal children, bone-related muscle area is the main determinant of prepubertal diaphyseal bone size, strength and bone mineral content (BMC) [29]. In healthy Chinese children aged between 5 and 19 y, total and appendicular lean mass explained between 85 and 95% of the variation in whole-body-less-head BMC and BMD [30]. The observation during puberty that the peak velocities (pv) of accrual of lean body mass precedes that of bone mineral content is also consistent with the mechanostat [31,32,33,34,35]. Because there are gender differences in the bone–muscle relationship observed during puberty and old age, it has been suggested that age- and gender-related variations in both endocrine regulation and molecular signalling between bone and muscle that are independent of the purely mechanical interactions, also influence the relationship [36].

While muscle growth during normal postnatal development has been much less discussed in the context of the mechanostat, there is ample evidence for the mechano-sensing of myocytes [26,37,38,39]. This means that the growth of the appendicular muscles in response to the corresponding appendicular bone length growth through passive stretch, is an obvious mechanism since it has long been known that muscle responds to passive overstretch through sarcomerogenesis, the creation and serial deposition of new sarcomere units of myofibrils [40]. Teleologically the growth in muscle size and strength relative to its associated bone must be sufficient for the movement in which it is involved and in practice, such growth is a function of both bodyweight and especially limb length, i.e., bone length. In fact, muscle mass increases at the 3rd or 4th power (depending on the muscle position), of the increases in length of the associated long bone [7,41] allowing a proportionality between strength and body weight to be maintained. Furthermore, all studies of pubertal growth patterns have shown pv height to precede pv lean body mass [32,33,34], or, when only yearly measurements of height and lean body mass, (LBM), are recorded, in the same year [42].

The clearest evidence for this is a detailed longitudinal study of bone and muscle growth throughout puberty in a cohort of 11-year-old healthy Finnish girls [43]. Bone size and mineralisation and muscle cross-sectional area (CSA) was measured by peripheral quantitative computed tomography (pQCT), and tibial length was measured from DXA scans. Growth trends as a function of time relative to menarche were determined over 7 years from prepuberty to early adulthood and the timing of peak growth velocities relative to menarche (on average at 12.9 years) are shown in Table 1. Whilst growth of all measures of bone size and mineral density and muscle were rapid before and after menarche and had not completely achieved adult values at the end of the 7-year follow up, there was a clear hierarchy of peak growth velocities before and up to menarche. Thus, tibial length and CSA growth velocities each peaked 20 mo before menarche with their growth essentially completed 2 years after menarche. Muscle growth followed, peaking 1 year later, with bone cortical thickness and mineralisation peaking over the following 8 months. These measures continued to increase, so that at the age of 18 years, they were still significantly lower than adult values. The growth sequences of bone dimensions and mineralisation and muscle size resulted in a relatively constant calculated bone–muscle strength index, a measure of the balance of bone strength to the load on it from muscle contraction, from prepuberty to early adulthood.

**Figure 1:**
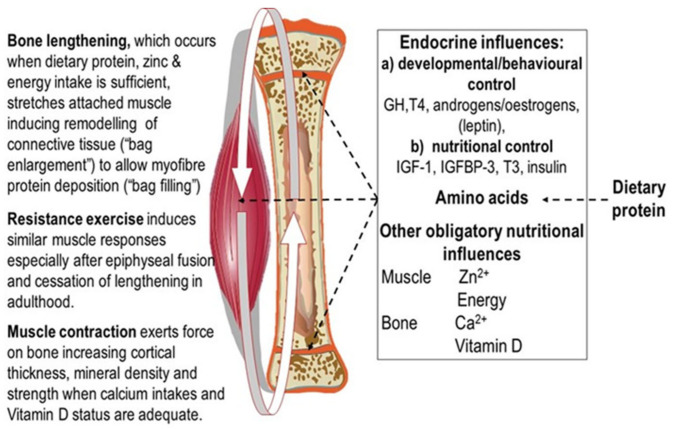
Dietary protein and appendicular muscle- bone interactions.

The simplest explanation of these growth patterns is as postulated in the Protein-Stat model, i.e., bone growth in length is the independent and primary determinant of whole-body growth, controlling muscle growth through a passive stretch mechanism [7], which in turn acts on bone through the mechanostat to increase bone mineral density and strength. Thus, muscle and bone growth are intimately connected in a bidirectional relationship, (shown in Figure 1).

As a result of this relationship, muscle mass, as indicated by muscle strength in children, is highly dependent on height, which accounts for about 80% of its variation [44], at least up to the age at which muscle mass in boys starts to exceed that in girls. In healthy Chinese children, while height-adjusted DEXA-assessed total body and appendicular lean mass was slightly higher in boys from the age of 5, the difference markedly increased during the adolescent growth spurt from the age of 13 years [30], as did both grip strength [45] and the strength of many individual muscle groups [46]. Clearly the muscle mass–bone length relationship will be modified by the endocrine factors which mediate the differential muscle growth between adolescent males and females.

The control of the growth of the major appendicular muscles in childhood is directly related to the lengthening of the associated bone which occurs by endochondral ossification in the growth plate. This latter process is regulated through endocrine and nutritional influences, especially that of dietary protein and by a paracrine/autocrine system of bone growth factors [47,48] within which amino acids and zinc have direct roles in signal transduction [49,50,51,52,53], although the specific sites of action in the growth plate have yet to be described. Bone lengthening stretches muscle, activating mechanosensitive pathways [54,55] at the cell–matrix interface [56,57] which results in activation of satellite cells to add myonuclei to myofibres which increase protein synthetic capacity in terms of ribosomal RNA [58] enabling increased myofibre protein synthesis [59,60]. In addition, fibroblasts are activated to increase collagen synthesis [61,62,63] which enables remodelling of the ECM, changes observed during the stretch induced growth of a wing muscle in the fowl many years ago [64]. Bone osteocytes are mechanosensitive and their mineralisation is responsive to mechanical forces exerted by muscle [26]. Thus, muscle and bone growth are intimately connected in a bidirectional relationship in which bone length growth regulates muscle mass and muscle growth regulates bone strength.

### 2.3. Muscle and Bone Growth Interrelationships during Saltatory Growth

Most of what we know about bone growth derives from a variety of small animal and organ culture studies where growth is rapid. However, an important feature of human growth is that it is very much slower, falling rapidly from a rate of about 0.6–0.75 mm/day during the first year to a nadir of 0.15 mm/day prior to puberty when it peaks at about 0.27 mm/day in boys and 0.22 mm/day in girls [42,65]. The detailed time course is subject to some debate in terms of whether it is continuous or episodic as well as whether the concept of canalisation is valid [66], although there does seem to be a uniform pattern of growth during the pubertal growth spurt in terms of the timing of the peak height velocity in relation to final height [67].

The question of whether growth is continuous or episodic is important in terms of identifying nutritional drivers of growth. One longitudinal growth study was reported to display a number of spurts of regular occurrence throughout the prepubertal period, i.e., a cyclical pattern of growth [68,69]. The most important (and contentious) phenomenon is that length growth only occurs in short bursts: i.e., it is saltatory, (discontinuous with leaps), with 90–95% of infant development growth-free [70,71,72,73]. This phenomenon is said to occur throughout development, although with fewer, more prolonged bursts in older children [74,75]. The timing of growth of muscle in relation to these saltatory increases in bone length has not been specifically defined. Weekly measurements of weight, length and skinfolds in infants in the first year showed a saltatory pattern for both length and weight gain with weight and length growth coupled in males and females although the timing of the coupling differed somewhat between sexes [73] and muscle or lean body mass growth as opposed to adipose tissue growth was not specifically estimated. Measurements of weekly gains in height, weight and overnight urinary growth hormone output in healthy prepubertal school children over nine months, demonstrated synchrony between height and GH, weight and GH, and especially height and weight [76]. It could be argued that the phenomenon of saltatory length growth, at a faster than average rate, is necessary to generate sufficient passive-stretch force on muscle to induce muscle growth, but this has not been investigated. On the basis that muscle growth is controlled by the mechanotransduction in response to stretch from bone lengthening then the time course of that linkage is an important question to be resolved. Indeed, whilst the “growing pains” experienced in the lower limbs of children [77] have in the past been postulated to be a result of rapid bone lengthening and associated stretching of muscles, such ideas do not have widespread support amongst paediatricians and are seldom listed as a potential aetiology [77].

One exception to the usual slow growth of infants and children is catch-up growth in weight and muscle mass during recovery from muscle wasting due to malnutrition. In this case, after suitable treatment the appetite is markedly stimulated and the rate of weight and muscle growth can be remarkably rapid [78], limited only by the rate at which the child can consume food energy containing sufficient type 2 nutrients (e.g., protein and zinc [79]). However, when the normal weight-for-height is achieved, appetite and food-intake return to normal, and growth in weight slows markedly to a near-normal rate for age and height. Growth in length restarts after most of the weight deficit has been achieved with the extent and pattern of catch-up in height varying with the environment [65,80].

## 3. Endocrine and Cellular Mechanisms of Growth in Stature during Development

### 3.1. Endocrine Regulation of Statural Growth

As shown in Figure 1 endocrine influences on bone and muscle growth involve those under developmental/behavioural control, i.e., growth hormone (GH), thyroid hormone, androgens/oestrogens and possibly leptin and those deemed to be under nutritional control, IGF-1, insulin-like growth hormone binding protein 3, (IGFBP-3), tri-iodothyronine, (T3), and insulin, together with inhibitory factors such as cortisol and fibroblast growth factor 21, (FGF21), [81,82], making for an extremely complex system.

The IGF-1 gene consists of six exons and gives rise to several mRNA variants all of which yield a common 70 amino acid peptide required for activating IGF-1 receptors but differ in terms of tissue of expression and whether they contribute to the circulating IGF-1 pool. Liver expresses Class 1, Class 2, Ea and Eb transcripts and contributes to 75% of the circulating IGF-1 level while skeletal muscle IGF-1 transcripts initiate from exon 1 (Class 1 variants). IGF-1 in circulation has a very short half-life and its action is regulated through the IGF binding proteins especially IGFBP-3. In early infancy, IGF-1 circulates in a binary complex with IGFBP-3 but once GH control of IGF-1 is established probably towards the end of the first year of life, IGFBP-3 maintains a circulating ternary complex with IGF-1 and the acid-labile subunit (ALS), a circulating hepatic-derived glycoprotein, prolonging the half-life of bound IGFs and forming a long-lasting reservoir of IGFs in the circulation [83]. This means that unlike insulin, serum IGF-1 is less influenced by the immediate fed-state.

It is generally accepted that GH and IGF-1 are the main primary drivers of length growth [84,85,86] but in the foetus and early infancy, GH may be much less involved, with IGF-1 and insulin playing the major role. In the Karlberg infancy–childhood–puberty (ICP) growth model of three additive and partly superimposed components of linear growth, this infancy component is proposed to last for about 10 months [11,87,88].

In fact, in the postnatal growth plate, insulin, GH and IGF may have overlapping functions. There can be crosstalk between IGF-1 and insulin at receptor level [89] and post receptor signalling via the Ras/mitogen-activated protein, (Ras/MAP), kinase pathway is identical [90,91]. Additionally, although the GH receptor is distinctive, some of the GH- Janus kinase 2 (JAK2)-initiated signalling is similar to insulin/IGF-1 post receptor signalling [92,93]. Because the GH receptor is found on chondrocytes of all zones of the growth plate [94], GH action is more complex than simply activating expression of IGF-1, Bone morphogenetic proteins, (BMP), and other genes in resting zone chondrocytes through the JAK/STAT pathway. In addition, IGF-1, like GH, can stimulate proliferation of resting zone chondrocytes and chondrocyte hypertrophy [95].

IGF-1 is expressed in tissues throughout the body and its role in height growth is likely mediated mainly through paracrine/autocrine mechanisms within the growth plate [84,86]. However, in IGF-1 transgenic mice, which overexpress the hepatic IGF-1 gene with high levels of serum IGF-1, weight and length growth is either increased in otherwise normal mice (which express growth plate IGF-1), or normalised in IGF-1 KO mice (apart from some early postnatal skeletal growth slowing) [96]. This shows that at high plasma levels, IGF-1 is able to mediate growth to some extent in an endocrine fashion in the absence of any para/autocrine effects.

In infancy, insulin is proposed as primary regulator of hepatic IGF-1 generation in both the foetus and the newborn infant, so that effects of nutrients, such as glucose and protein, can influence IGF-1 through insulin [97]. Insulin regulates hepatic GH receptor availability [98] and enhances the GH signalling pathway to enable hepatic IGF-I production in the fed state and increases circulating IGF-1 levels [84]. GH appears to play a minor role, if any, in the regulation of intrauterine and immediate postnatal growth, with its main role involving the regulation of glucose homeostasis together with cortisol [84,99]. Sleep is not thought to be a stimulus for GH secretion until 3 months of age, but feeding and insulin are known to stimulate GH secretion [100]. Full control by GH of the hepatic IGF-1, IGFBP3 and the acid labile subunit (ALS) genes, is developmentally regulated and fully established towards the end of the first year of life at the beginning of the childhood growth stage [84,97,101]. Insulin levels are reported in a minority of studies examining IGF-1 and growth, and in most cases fasting levels are reported, which do not reflect postprandial levels likely to be the main influence on IGF-1 production. It may be that much of the variation in clinically reported IGF-1 levels (see Section 4 below) reflects differences in insulin response to food, and that IGF-1-related growth responses, especially in relation to adipose tissue growth, reflect insulin rather than IGF-1-mediated effects. Certainly insulin levels and urinary C-peptide concentrations are very much lower in breastfed compared with formula-fed infants in line with the lower IGF-1 levels [102,103], while in 8-year-old boys the increased IGF-1 levels in response to an increase in milk but not meat intake [104], are associated with higher insulin levels [105].

The activity of the insulin–GH–IGF axis is dependent on the other endocrine axes, which have permissive actions on GH-stimulated IGF-I expression and affect IGFBPs and proteases. The thyroid axis is vital for normal growth [106] and the maintenance of bone [107]. T4 acts to enhance GH effects on endocrine IGF-I expression and conversely a normal GH–IGF–IGFBP axis is needed for optimal thyroid hormone production [108]. Both androgens and oestrogen contribute to the pubertal growth spurt [81,109,110]. Androgens can stimulate longitudinal bone growth acting directly on growth plate chondrocytes and indirectly by increased local IGF-I expression. Androgens also act in boys after aromatisation to oestrogens which signal through the ER-α receptor to mediate the growth spurt directly and, like androgens, through stimulation of the GH–IGF-I axis.

Whilst these general aspects of the endocrine and paracrine/autocrine regulation of bone length growth are generally agreed, there are several difficulties relating this framework to an understanding of the control of human growth.

Firstly for infants and young children the possibility that length growth is saltatory and under the control of growth hormone acting on the growth plate which in turn is regulated by factors such as sleep [72,111], means that the role of those nutritionally regulated endocrine factors, IGF-1, T3 and insulin which might be modulated by dietary protein is difficult to determine with any certainty, although the stimulation of appetite in response to a saltation affords an obvious opportunity for nutritionally related endocrine regulation. There are no reports of any attempts to relate changes in IGF-1 or nutritional intakes to the extent or periodicity of saltations and most studies of nutrition, IGF-1 and growth would not allow this to be done. In fact, almost all paediatric studies of growth ignore the possibility of saltation.

Secondly the relationship between the most widely measured hormone factor, circulating IGF-I levels, mainly a measure of hepatic production, and IGF-1 levels in the growth plate or in muscle, produced and acting in an autocrine/paracrine fashion cannot easily be clinically defined. Both hepatic IGF-1-deficient mice and ALS knockout mice exhibit relatively normal growth and development, despite having 75% and 65% reductions in serum IGF-1 levels, respectively. However, loss of both genes which further reduced serum IGF-1 levels, did significantly reduce linear growth [112,113]. Human mutations which influence hepatic IGF-1 or binding protein or ALS [114] dramatically reduce serum IGF-I concentrations but do not result in significant growth impairment in contrast to tissue-wide IGF-1 gene deletions [115]. Yaker et al. [112] suggest that a threshold low concentration of circulating IGF-1 is necessary for normal bone growth, and as discussed above markedly increased hepatic IGF-1 production and serum levels can increase growth or normalise it in extra hepatic tissue igf1 gene KO mice [96]. More complexity is suggested by observations that systemic IGF-I is necessary to maintain cortical bone structure, whereas skeletal IGF-I appears to play a more significant role in the maintenance of trabecular bone [85].

Finally, the most clinically relevant measure of circulating IGF-1 activity, (i.e., total IGF-1, free IGF-1, the IGF-1-IGFBP-3 ratio or IGFBP-3), is by no means agreed and each of these values are reported in different studies. About 1% of the total serum IGF-1 is free and can be measured by a specific immunoradiometric assay and some have speculated that total IGF-I concentration does not reflect the IGF-mediated bioactivity of the serum entirely, because IGFBP concentrations as well as IGFBP proteolytic activity may modify the bioavailability of IGF to the tissues. In this case, the free unbound IGF-I may represent the biologically active fraction of the total circulating IGF in analogy with thyroid and sex steroid hormones. In a cross-sectional study of Danish children [116], free IGF-I serum levels increased during childhood, with highest values in puberty, 1–2 years earlier in girls than in boys. These peak values occurred approximately 1 year later than a previously reported age of peak height velocity. The authors argued that this finding does not support their hypothesis that free IGF-I by this assay better reflects longitudinal growth in children than total IGF-I, a conclusion recently re-emphasised [84]. Indeed, it has been argued that because IGF-II also binds with IGF-BP3 and ALS in the ternary complex and because the IGFs and IGF-BP3 are rapidly cleared from the circulation outside of the ternary complex, calculating the molar ratio of total IGF-I to IGFBP-3 as an index of IGF-I bioavailability, disregarding IGF-II concentrations, does not make any sense [84]

Thus, these caveats must be taken into account when trying to interpret the paediatric literature on dietary protein and growth in stature and muscle mass discussed below in Section 4.

### 3.2. Endochondral Ossification

**Figure 2:**
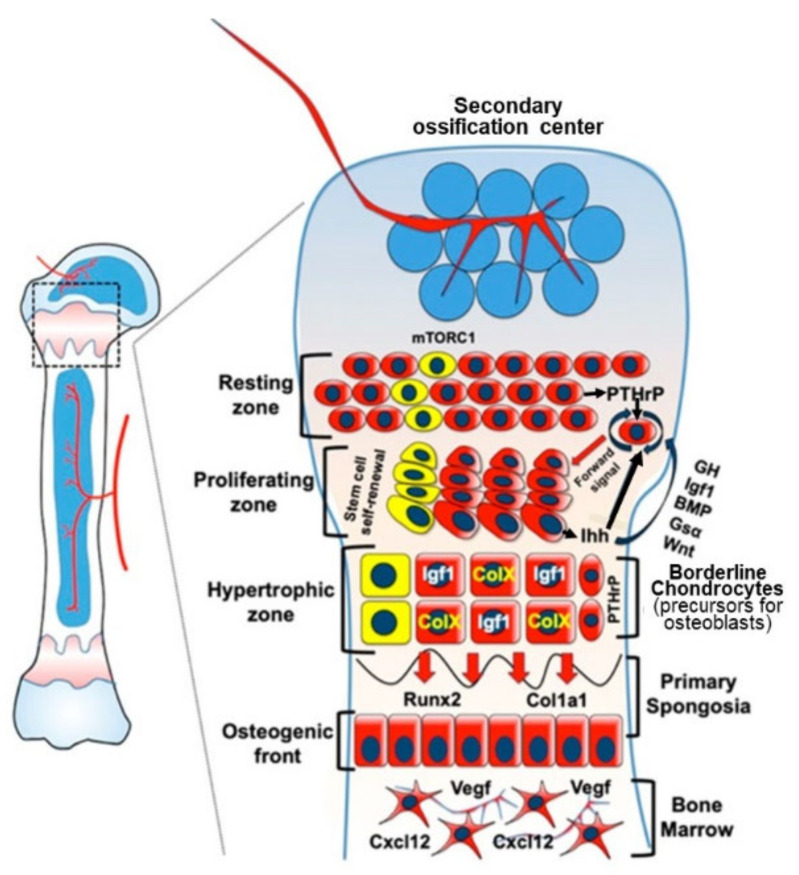
Endochondral ossification: molecular characteristics of growth plate chondrocytes and their differentiation trajectories. Stem cells in the resting zone express the transcription factor SOX9 which drives their commitment and differentiation into chondrocytes. The initial development of the secondary ossification centre stimulates the expression of parathyroid hormone-related protein (PTHrP) by skeletal stem cells in the resting zone niche. This population contributes to the formation of proliferating and hypertrophic chondrocytes in addition to marking collagen Type I Alpha 1 Chain, (Col1a1+), osteoblasts in the primary spongiosa and stromal cell-derived factor 1, (Cxcl12), expressing bone marrow stromal cells in the marrow cavity over time. PTHrP acts to delay differentiation and keep the chondrocytes in a proliferative state, becoming organised into clones that arrange themselves into columns, contributing to bone elongation via interaction with adjacent cells. However, when these cells become sufficiently distant from the source of PTHrP due to interstitial growth, they differentiate into pre-hypertrophic and early hypertrophic chondrocytes, which are postmitotic cells that express Indian hedgehog (Ihh). This factor diffuses up the column to stimulate further PTHrP secretion to maintain the proliferative nature of the chondrocytes. After terminal differentiation, chondrocytes enlarge and this is the major contributing factor regulating the growth rate in endochondral bones. mTORC1 activity maintains the stem cells in their niche and remains important throughout proliferation and hypertrophy. Growth hormone acts within the resting zone to increase expression of IGF-1 and its receptor which is important for the PTHrP-Ihh feed-back loop and for the process of hypertrophy. T3 (not shown) is widely involved with indirect effects involving enhancing IGF-1 action and directly inhibiting proliferation and stimulating pre-hypertrophic and hypertrophic chondrocyte differentiation [120]. Bone morphogenetic proteins (BMPs) and their receptors are expressed in proliferating and hypertrophic chondrocytes and BMP signalling regulates chondrocyte proliferation and differentiation in part within the PTHrP-Ihh feed-back loop and by maintaining SOX9 activity. Gsα is a GTPase which activates adenyl cyclase to increase cyclic AMP and mediates the action of PTHrP and inhibits Ihh. Wnt(Wingless-related integration site signaling glycoproteins)/β-catenin signalling promotes differentiation of proliferating chondrocytes into hypertrophic chondrocytes both by antagonising PTHrP and independently of PTHrP signalling. Copied and adapted from Hallett et al. (2019) [48] under the Creative Commons Attribution 4.0 International license.

The target of the anabolic drive for linear growth of bones, which directly influence height, (limb bones, ribs and vertebrae), is endochondral ossification [48,117,118,119] (see Figure 2). This involves a chondrocyte differentiation cascade initiated by stem cell clonal expansion as proliferative chondrocytes, followed by hypertrophy, cartilage matrix secretion and apoptosis which releases angiogenic factors that stimulate vascular invasion and migration of osteoblasts and osteoclasts, leading to remodelling of calcified cartilage and formation of trabecular bone [47,117]). Endochondral ossification occurs both in the growth plate to enable longitudinal growth of the metaphysis (primary ossification) and in the epiphysis to form the head of the bone (secondary ossification). It is regulated by an extremely complex system involving the combined influence of systemic endocrine and local paracrine/autocrine anabolic influences [47,48,82,119,120] together with nutritional influences on signal transduction.

While the endocrine anabolic influences of GH and IGF-1 [86] and thyroid hormones [107] and the local paracrine/autocrine influences on endochondral ossification are relatively well understood, as discussed elsewhere [120], the extent and nature of direct anabolic influences of amino acids and important regulatory cations like Zn^2+^ on endochondral ossification are poorly understood. However, chondrocyte development in cell culture is sensitive to amino acid levels [121], length growth in vivo in rodent models is extremely sensitive to dietary protein intake, and given the importance of mTORC1 for bone growth [122,123,124]) and for the coordination of chondrocyte proliferation and hypertrophy [78] as shown in Figure 2, there is no reason not to believe that amino acids activate it as identified in other tissues. It is the case, however, that mTORC1 activation in relation to its interaction with the lysosome [125], may be different in chondrocytes [126]. The relationship between dietary protein intake and linear growth in infants and children is discussed below in Section 4.

As for zinc, although it is clear from several animal studies that adequate zinc is required for normal bone growth [49] and that the cellular and molecular role of Zn^2+^ in mediating signalling cascades via a variety of pathways is becoming better understood [50], the specific sites of action in the growth plate are only gradually emerging. Zn^2+^ has recently been shown to induce expression of both chondrogenic genes, the transcription factors SRY-Box 9 (Sox9), runt-related transcription factor 2 (Runx2) and collagen) as well as genes associated with pro-angiogenic vascular endothelial growth factor, (VEGF),-mediated signal transduction [52]. The Zn^2+^ cation also promotes chondrocytes in ATDC5 cells, a highly proliferative cartilage cell line, to differentiate [53]. This is consistent with reports that disruption of the Zip family of Zn transporters induces growth retardation in animals and is causal to some human syndromes is associated with short stature [120].

## 4. Cellular Mechanisms of Muscle Growth during Development

### 4.1. Myofibre Hypertrophy within the Extracellular Matrix: The Nature of the Problem

Muscle growth at its simplest is muscle protein deposition, the balance between protein synthesis and proteolysis, and the regulation of this process is central to any understanding of protein nutrition and whole-body protein metabolism. However, the highly structured architecture essential to muscle function makes it amongst the most complex organs of the body, and describing the regulation of its growth in any detail in cellular and molecular terms is challenging given its structure which is necessary for its function.

At birth, skeletal muscle comprises multinucleated myofibres which have derived from embryonic and foetal precursor myoblasts and myotubes. On the basis of mainly animal studies, postnatal muscle myofibre growth involves largely hypertrophy, i.e., increases in their length and cross-sectional area (CSA), [127,128], since the consensus is that, in human muscle, there is little or no expansion of their number (i.e., hyperplasia) during normal growth [129,130].

**Figure 3:**
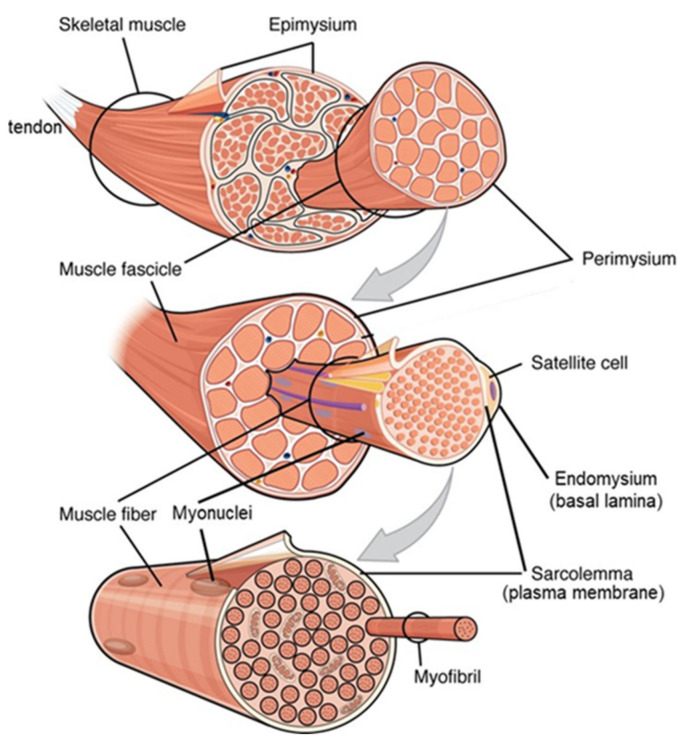
The three connective tissue layers or “bags” which define the volume of an individual skeletal muscle. The entire muscle, (**top**) is encased by the epimysium. Bundles of muscle fibres, (fascicles, **middle**), are encased by the perimysium. Each muscle cell, (**middle** and **bottom**), the multinucleate myofibre containing the myofibrillar contractile apparatus, is bounded by the sarcolemma or plasma membrane which is encased by the endomysium or basal lamina. Muscle stem cells (satellite cells) reside outside the myofibre between the plasma membrane and the basal lamina. Copied and adapted under the Creative Commons Attribution 4.0 International license from [132,133].

Muscle’s function requires a structure which allows force transduction to be transmitted to its bone levers and as a result its myofibres insert directly onto bone or indirectly via aponeuroses and tendons [131]. These junctions include connection to the extracellular matrix (ECM) of connective tissue which encases each myofibre, (the endomysium or basal lamina), the fascicles (bundles of myofibres encased in the perimysium), and the entire muscle (epimysium), (see Figure 3), structures which define myofibre and muscle volume and carry the majority of muscle passive load [128].

Muscle growth involves myofibre hypertrophy through both protein deposition within the expanding myofibres as well as remodelling of their encasing ECM. The Protein-Stat deals with this with a simple “Bag Theory” of muscle growth and maintenance, originally introduced in 1988 [134]. Thus, muscle is conceived simply as a series of connective tissue “bags” encasing individual myofibres, fascicles and the entire muscle which volume-limit the myofibre and indeed the whole muscle, (see Figure 3). In this context, muscle growth involves ECM remodelling to enable “bag enlargement,” with muscle stem cells, (satellite cells, SC), proliferating and differentiating to provide new myonuclei to allow myofibre growth in size through “bag filling”, i.e., protein deposition as myofibre proteins. Normal growth in childhood would involve periods of bag enlargement associated with length growth, terminating soon after the end of adolescence when bone length growth stops. “Bag filling” is limited by the maximum myofibre volume within the myofibre basal lamina at which point a “bag-full” signal terminates myofibre protein deposition. Thus, bag filling and the bag-full state is an inherent feature of regulation of muscle maintenance during the diurnal cycle of feeding and fasting when postprandial protein-deposition repletes muscle protein lost during the postabsorptive phase. In childhood there is growth and maintenance whereas after the end of adolescence there is only maintenance. It is also possible that part of the repletion of muscle mass lost during malnutrition as occurs during the rapid catch-up growth involves bag filling as well as bag enlargement until such rapid growth ceases when children reach their expected weight for height and resume normal slow growth (see below) [78].

Muscle growth involves increases in myofibre length [135], through the addition of sarcomeres at the end of the myofibre [40], and an increase in girth [127,130,135]. As indicated above such fibre hypertrophy requires both the acquisition of new myonuclei within the myofibre and substantial remodelling of the ECM, which must be finely controlled to avoid overproduction and fibrosis. Because muscle is highly irrigated by blood vessels which encase each myofibre with a network of microcapillaries [136], during muscle growth, angiogenesis must also occur in a coordinated way to ensure the enlarged myofibre is adequately oxygenated and supplied with nutrients. How all this is regulated during human postnatal muscle growth is by no means understood but there is an extensive set of literature on the molecular mechanisms likely to be involved deriving from a variety of human and animal muscle cell studies and animal models of postnatal muscle growth, hypertrophy and regeneration after injury and it can only be assumed that similar mechanisms are in involved in human postnatal muscle growth, as discussed below.

### 4.2. Role of Satellite and Other Cells in Myofibre Hypertrophy

A recent analysis of the cell types and their transcriptomes in adult mouse skeletal muscle [137] indicates that in addition to the multinucleated myofibre, 10 additional mononucleated cell types are present comprising immune cells, endothelial cells, glial cells, connective tissue cells, and satellite cells (SCs). Of these SCs are the unipotent muscle stem cells, most obviously the source of new myonuclei in the growing muscle fibres. However, these other cells, often called mesenchymal stromal cells, include other populations of potentially myogenic cells which are molecularly and anatomically distinct from satellite cells, [138,139], which include pericytes associated with blood vessels [140,141], and several types of interstitial cells including PW1+/Pax7– interstitial cells (PICs) [142] and cells expressing the Twist2 (Tw2) transcription factor [143]. Other pluripotent progenitor cells lacking myogenic potential are fibro-adipogenic progenitors (FAPs) [144] which are important in the maintenance and growth of the extracellular matrix (see below). Whilst the precise role of some of these cells in postnatal human muscle growth is less understood compared with SCs, it is clear that the postnatal muscle is well equipped with a support group for its stem cell-mediated growth [138,145] in terms of forming new myofibres, associated connective tissue, tendons, blood vessels and nerves. It will be assumed here that during postnatal maturational skeletal muscle growth, satellite cells do play the principal role of mediating myonuclear accretion within the myofibre to support the additional transcriptional demands of protein synthesis associated with postnatal development and protein turnover in the growing muscle myofibre. Certainly in the mouse there are significant increases in mouse myofibre size and myonuclear number between prepuberty and early adolescence/puberty onset (4–6 weeks of age) and experimental prepubertal depletion of SCs reduced myofibre size and myonuclear number, and caused force generation deficits) [146]. However, as discussed below, there seems to be a phase of myofibre enlargement during the post pubertal growth phase in the adult rat with fewer additional myonuclei added so that the connective tissue remodelling needed for “bag enlargement” at this time must be achieved by achieved by the mesenchymal stromal cells which support SCs.

**Figure 4:**
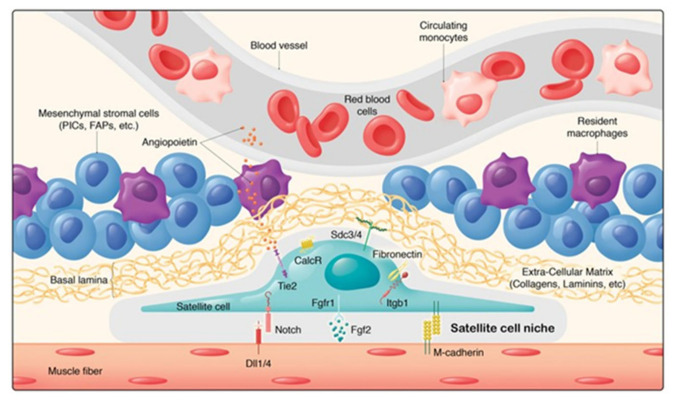
The satellite cell and stromal cell niche [139]. Satellite cells states are regulated through their interactions with their microenvironment. While direct interactions (M-cadherin, Notch pathway) and communication (FGF2/FGFR1 pathway) between muscle fibres and satellite cells have been identified, muscle stem cells also interact with a variety of components of the extracellular matrix (e.g., Collagens VI and V, Laminin, Fibronectin, SDC3/4) and diffusible cytokines and growth factors (e.g., Angiopoietin-Tie2 receptor). In addition to satellite cells, several cell types contribute to muscle growth, homoeostasis and regeneration, including pericytes, mesenchymal stromal cells (e.g., Pw1+ Interstitial Cells, Fibro Adipogenic Progenitors, Twist2+ cells) immune cells (e.g., resident or infiltrating macrophages) as well as connective tissue cells. Copied under the Creative Commons Attribution 4.0 International license from Evano and Tajbakhsh, 2018 [139].

Satellite cells (SCs), in common with all tissue stem cells, are usually located in a specific microenvironment or “niche” between the myofibre sarcolemma and the basal lamina (see Figure 4 [139]). SCs can receive an array of spatially discrete and temporal cues, both structural and biochemical, emanating from this microenvironment [26,38,139,147,148,149,150]. Within this, niche SCs are also in contact with endothelial cells (ECs) of the microvasculature [151] and there is a proportionality between SC number and the degree of capillarisation of the associated myofibres [152]. ECs secrete IGF-1, HGF, FGF, PDGF, and VEGF, and coculture of SCs with ECs promote SC proliferation, which is blocked by inhibition of the growth factors [151]. Direct contact with ECs of the microvasculature is achieved via vascular endothelial growth factor A (VEGFA) which is expressed by SCs and which can bind to VEGFA receptors on capillary ECs. Subsequent crosstalk between ECs and SCs via EC-derived Notch ligand Dll4 and the Notch signalling pathway in SCs is vital for replenishment and maintenance of quiescent satellite cells [153].

**Figure 5:**
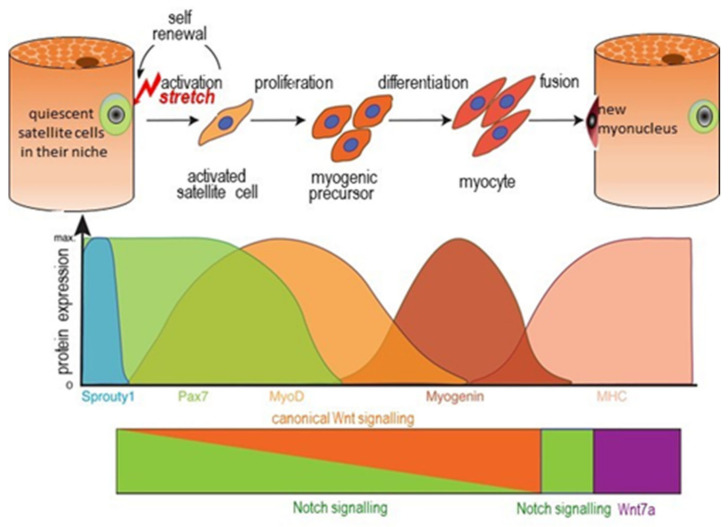
Myogenic lineage progression and expression profile of key myogenic regulators [155]. **Top** Schematic illustration of the myogenic lineage progression. Satellite cells in their niche are activated by overstretch, start to proliferate, thereby generating myogenic progenitor cells. Upon differentiation, myogenic progenitor cells differentiate into myocytes, which fuse with the myofibre to form new myonuclei enabling myofibre hypertrophy and muscle growth. **Middle** Expression profile of key modulators of myogenic lineage progression. Sprouty 1 expression maintains a mitotically inactive state similar to quiescence [156]. The transcription factor Pax7 is required for the postnatal maintenance of satellite cells (SCs) and to enable their activation, but is lost after activation and proliferation and it cannot be identified in myonuclei after fusion with the myofibre [157,158]. Of the main myogenic regulatory factors (Myf5, MyoD, myogenin and Mrf4), MyoD is an early marker for myogenic commitment and Myogenin is a direct target of MyoD and initiates the terminal differentiation of myogenic progenitor cells, which is accompanied by downregulation of MyoD expression. The terminally differentiated myocytes fuse with the myofibre and express muscle genes such as myosin heavy chain (MHC). **Bottom**. A switch from Notch to Wnt signalling is required for proper satellite cell differentiation. Satellite cells express high levels of Notch to retain them in a quiescent state and upon activation canonical Wnt signalling increases. Canonical Wnt signalling—mainly through the ligand Wnt3a—drives differentiation of satellite cells, while non-canonical Wnt signalling through the ligand Wnt7a is responsible for promoting symmetric satellite cell divisions, migration of satellite cells, fusion with and growth of myofibres. Upon return to quiescence, satellite cells switch to Notch signalling. Copied and modified from Schmidt et al., 2019 [155] under the terms of the Creative Commons Attribution 4.0.

SC quiescence is a specific state in that during homoeostasis, satellite cells are maintained in a reversible non-proliferating quiescent (G0) state by a number of regulators [139,154] and can cycle into a G(alert) state in response to systemic factors with increased proliferative capacity and regenerative potential. It is not clear whether, during the postnatal periods when no growth is occurring, SCs exist in the quiescent (G0) or G(alert) state. Nevertheless, we must assume that when it occurs, bone length-growth induces muscle stretching, activating the SC myogenic sequence enabling myonuclear addition to myofibres during postnatal growth (Figure 5 [155]). With different signals on the basal and apical surfaces of SCs, intracellular signalling is polarised resulting in cell division being either symmetric, expanding the stem cell population or asymmetric leaving one daughter in contact with the basal lamina to repopulate the niche, and the other in contact with the myofibre which becomes a committed myogenic progenitor cell. This then differentiates into a myocyte which can fuse with the myofibre and add a new myonucleus beneath the sarcolemma.

SC activation and transformation to a new myonucleus necessitates a change from a very limited transcriptome to the fully extended one of the terminally differentiated myonucleus within myofibres. The processes of proliferation and differentiation are mutually exclusive, and this transition is mediated by transcription factors, proteins that bind to DNA and regulate gene expression by promoting or suppressing transcription [159,160,161,162], (see Figure 5), working in concert with epigenetic regulators [163]. Sprouty 1 expression maintains a mitotically inactive state similar to quiescence [156]. Additionally, caveolin-1 a marker protein for caveolae invaginations of the SC plasma membrane is associated with satellite cells in a more quiescent state [164]. The MyoD (myoblast determination protein) family of four myogenic regulatory factors, (MRFs), include Myf5, (myogenic factor 5), and MyoD, early markers for myogenic commitment which commit cells to the myogenic program, myogenin, a direct target of MyoD, and Mrf4(myogenic regulatory factor 4), each of which control expression of the terminal differentiation genes. In fact, Mrf4, (not shown in Figure 5), the most abundant MRF expressed in muscle throughout postnatal development in all muscle fibre types, may play an important role in differentiated myocyte formation through its interaction with another transcription factor, myocyte enhancer factor 2, (Mef2) [165]. Mef2 appears to positively direct the expression of the differentiated muscle myofibre protein phenotype and thus must become active when SCs finally fuse with the myofibre to provide a new myonucleus. The suggestion from rodent Mrf4 knockdown studies [166,167] is that prior to fusion with the myofibre, activated SCs are held in check by Mrf4 which represses the final activation of muscle-specific genes by Mef2. This means that mechanotransduction may have to lift this inhibitory action of Mrf4 on Mef2. Adding complexity to SC activation during myogenesis is an additional regulatory layer involving MicroRNAs(miRNAs) [168,169]. These are small noncoding RNAs, which negatively regulate gene expression with the majority of human genes containing miRNA target sites. It would appear that upregulation of the expression of networks of muscle-specific miRNAs is achieved by MRFs and Mef2. One important example is miR-206 which is excreted in exosomes by activated SCs to be taken up by interstitial fibrogenic cells to control collagen biosynthesis and prevent excessive ECM deposition [170]. MiRNAs also regulate several signalling pathways including the IGF/AKT/mTOR pathway. Finally, another important transcription factor is serum response factor, (SRF), a member of the MADS-box transcription factor family which can activate target genes in association with coactivators, one of which in muscle cells is the myocardin-related transcription factor (MrtfA and B). SRF/Mrtf accumulate in the nucleus through G-actin-mediated mechanisms [171] and influence expression of the muscle-determining factor MyoD [172].

The final stage of the postnatal SC progression after activation involves fusion with the myofibre sarcolemma after membrane remodelling, hemifusion and pore formation. How this is achieved is poorly understood although SRF is thought to be involved in controlling the organisation of the actin cytoskeleton during myoblast fusion [173]. Studies in differentiated myoblasts suggest a mechanism for fusion involving two surface proteins: myomaker, a large multipass membrane protein which initiates membrane fusion, and a very small protein myomerger which generates membrane stresses to drive the transition to complete fusion [174].

The SC activation sequence involves interactions with a wide range of paracrine signals originating from the different cell types in and close to the niche shown in Figure 4 [138,139,150]. The regulatory interplay between SCs and immune cells, endothelial cells, fibrogenic cells, and adipogenic progenitors has emerged to be of particular complexity [153,175]. Reciprocal control of SCs and fibroblasts is indispensable for efficient expansion of the SC pool and for appropriate ECM protein production whilst keeping interstitial fibrosis in check [170,176,177]. Both fibroblast-like cells and adipocytes residing in skeletal muscle are derived from the common bipotent mesenchymal fibro-adipogenic progenitor cells (FAPs) [144]. FAPs share many molecular and functional properties with mesenchymal stem cells and, thus, many of their proposed functions in regeneration have been aligned with those of SCs. FAPs express and secrete IGF-1 and other growth factors which can participate in the activation of SCs [145]. The signals that stimulate SC activation and proliferation also activate FAPs which can proliferate and differentiate into fibroblasts a major cellular source of ECM proteins. The requirement for activation of FAPs is shown by studies in which FAP lineage decisions during regeneration are altered. This perturbs extracellular matrix (ECM) remodelling and impairs myogenesis [178].

The large numbers of SCs in human muscle in infancy, which decline as growth slows [129], is a phenomenon also observed in the initial phase of experimentally stretch-induced muscle hypertrophy in the adult fowl when there was a doubling of total muscle DNA (i.e., diploid nuclei) by day 3 [59] (see Table 2 below). Of these nuclei, the initially very small proportion of satellite cells is increased by orders of magnitude [179]. This suggests a possible initial overproduction of SCs and other extra-myofibre cell types, e.g., fibroblasts, perivascular cells, and immune cells, associated with the extensive remodelling activity during this phase of rapid postnatal growth. The mechano-dependency of skeletal muscle SCs was also demonstrated in stretched rat skeletal muscle in vivo, in which myonuclei number increased [180], and SC activation occurred, as indicated by increased myonuclei, myogenin and Mrf4 [37]. In isolated satellite cells, both proliferation and differentiation was upregulated in response to cyclic stretching [181,182].

### 4.3. The Mechanotransduction of Satellite Cell-Induced Muscle Growth

It is clear from the above that mechanotransduction subsequent to muscle stretch, coupled with paracrine and autocrine growth factor production within the SC niche from a variety of muscle resident and circulating cells, is able to link bone lengthening to muscle growth. The question considered here is the molecular basis for such mechanotransduction. At the outset it is important to recognise that the initiation of mechanotransduction- induced growth can occur for the most part independently from systemic factors other than those which mediate bone length growth, (i.e., mainly growth hormone, thyroid hormone and the sex hormones), the source of muscle stretching. This aspect of muscle growth was demonstrated in Goldberg’s studies of muscle hypertrophy in the 1960s [183,184] in which he showed in a synergist ablation model that compensatory hypertrophy occurred in hypophysectomised and even fasting rats. What this means is that mechanotransduction per se provides the initiating signal.

#### 4.3.1. Myofibre, Satellite Cell and ECM Linkages

**Figure 6:**
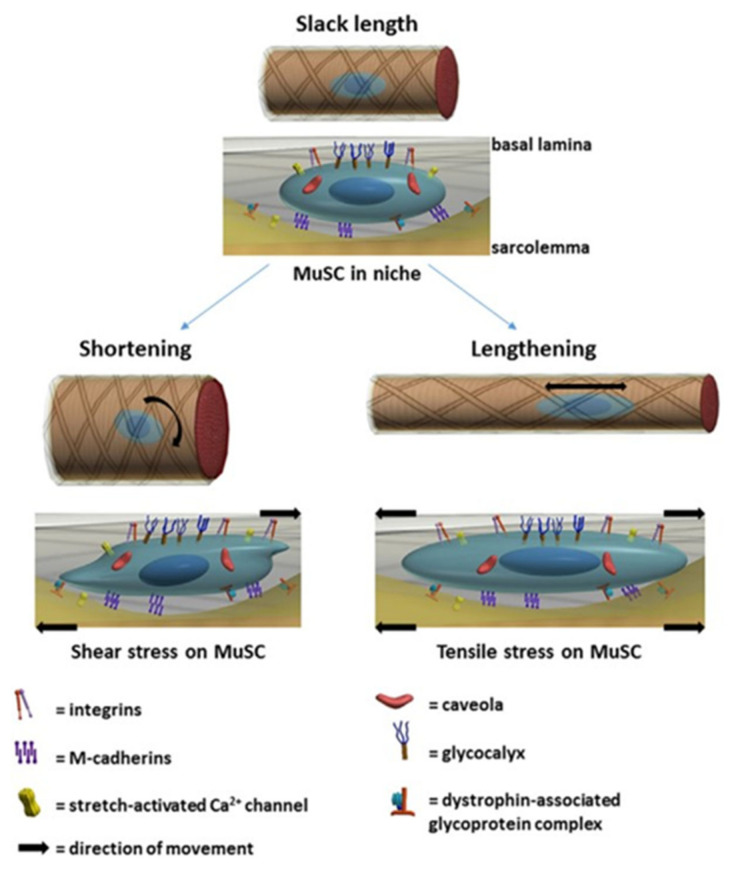
Potential effects of different types of stress on satellite cells. In response to mechanical overload by exercise or stretching, SCs are activated and proliferate to fuse with the host myofibre as shown in Figure 5. Both shear stress through shortening and tensile stress through lengthening may influence satellite cell orientation and deformation. In the unstrained (slack) myofibre is surrounded by a collagen fibre reinforced matrix (top). Below is shown an enlarged lateral view of the SC in its niche between the sarcolemma (yellow) and the basal lamina (BL). Transmembrane proteins anchoring the SC to the sarcolemma include M-cadherins and the dystrophin-associated glycoprotein complex. Both integrins and the glycocalyx anchor it to the BL. Caveolae are invaginations of the plasma membrane which function as message centres for regulating signal transduction and are identified by caveolin-1 which functionally regulates the activation state of caveolae-associated molecules, and which is associated with satellite cells in a more quiescent state [164]. With myofibre shortening, the sarcolemma will move relative to the BL and this is likely to twist the SC from a longitudinal orientation towards a more radial orientation (direction of arrow). As the myofibre is shortening, the sarcolemma and BL are likely moving at varying speeds, the relative movement of the sheaths will impose a shear force onto the SC, with subsequent cellular deformation. Myofibre lengthening will stretch both the sarcolemma and the BL (direction of arrow), which may induce a tensile stress on the SC. These physical cues, some acting through focal adhesion complexes, are thought to mediate the activation and differentiation of SC. Copied from Boers et al., 2018 [38] under the terms of the Creative Commons Attribution 4.0.

The structures involved in mechanotransduction are to be found within those involved with force production, the main function of muscle: i.e., the structures which enable force transmission from contracting sarcomeres within muscle fibres to the adjacent tendon and bone, i.e., inside-out transduction. This takes place both through the fibres themselves, as well as by lateral force transmission to adjacent parallel connective tissue network. Since individual skeletal muscle fibres do not extend throughout the entire length of a given muscle [130], the muscle depends upon its myofibre-ECM linkage for optimal function: i.e., an immediate linkage with the basal lamina, (BL), of the endomysium, as well as linkages through the perimysium [185]. Importantly in the present context, these connections for force transduction can be bidirectional, inside-out and outside-in, accommodating not only force transmission from the sarcomere of the contracting muscle to bone, but also stretch-induced tension on the whole muscle to the myofibre and its associated cells beneath the BL. Thus, when muscles and their myofibres are stretched SCs will be subjected to stress forces [26,38,55] as shown in Figure 6.

One linkage between the BL and the myofibre involves the highly specialised focal adhesion, (i.e., adhesive junction), the costamere, a large transmembrane macromolecular complex connecting the cytoskeleton of the myofibre directly to specific proteins within the BL. The main components of the costamere are the vinculin–talin–integrin system and the dystrophin-associated glycoprotein complex [186,187]. In the case of SCs, focal adhesions serve different purposes but nevertheless include macromolecular transmembrane structures connecting both the ECM and the myofibre to the cytoskeleton and nucleus of the SCs [38]. These include the gell-like glycocalyx layer, comprised of glycoproteins, proteoglycans, glycosaminoglycans and glycolipids which link the cytomembrane via glypicans or the cytoskeleton via syndecans to the ECM structure via glycosaminoglycan chains. This has mechano-transduction properties important for mechanosensing of shear stress [26].

Another important linkage involves the SPARC proteins (secreted protein acidic and rich in cysteine, osteonectin), extracellular matrix proteins that are regulatory but do not contribute significantly to ECM structure [188]. SPARC-like protein 1 (SPARCL-1) is secreted by human SCs [164] and has been shown to promote SC activation via focal adhesions.

The BL ensheathing the myofibre beneath which SCs reside in their niche, is composed of a network of ECM that directly contacts the satellite cell and the myofibre and separates it from the muscle interstitium. It is composed on the inside, next to the plasma membrane, of mainly a tight sheath of laminin-2 and on the outside next to the interstitium, of a mesh-like layer of collagen IV together with other collagens, fibronectin and other proteins, glycoproteins and proteoglycans.

The interstium, perimysium and endomysium comprises mainly collagen I and III [128,189]. These proteins are secreted by interstitial fibrogenic cells and by SCs at various stages of their development as they modify their immediate micro environment [150,190,191,192]. Satellite cells also signal to fibroblasts via miRNA-carrying exosomes to regulate ECM deposition during muscle remodelling [193].

#### 4.3.2. Molecular Mechanisms

Mechanotransduction of myofibre hypertrophy, myonuclear acquisition and ECM remodelling by muscle stretching requires signalling from the ECM and soluble factors to both existing myofibre myonuclei beneath the muscle plasma membrane and to SCs and associated cell types shown in Figure 4 within the interstitial space between myofibres. Ingber and colleagues [194] have reviewed mechanotransduction in the context of tensegrity (tensional integrity) architecture of cells: i.e., the mechanical stability of cells imposed by tensile prestress on the cellular cytoskeleton. The cytoskeleton of SCs, like that of all cells involves a complex network of interlinked biopolymers: actin microfilaments, microtubules, and intermediate filaments that determine the shape and mechanical stability of the cell, a dynamic structure undergoing continuous remodelling. Filamin A cross-links F-actin and anchors the cytoskeletal actin network to the cell membrane [187]. One important component of this cytoskeletal lattice is bundles of filamentous actin (F-actin) associated with myosin II, α-actinin and several other cytoskeletal proteins known as stress fibres (SFs) which are able to generate force [194,195]. The SC nucleus is stabilised by its own molecular skeleton or nuclear scaffold, and mechanical coupling between focal adhesions and the nucleus is mediated primarily by the cytoskeleton connecting cell surface adhesion sites to specific binding receptors on the nuclear surface that connect to internal nuclear scaffolds [196,197]. This is discussed further below.

The most important focal adhesions mediating the interactions of the dynamic ECM with the cytoskeleton of both myofibres and SCs are those formed by integrins and the dystrophin-associated glycoprotein complex (DGC) [198,199]. Cadherins, another major class of cell adhesion molecules are also involved in cell–cell “velcro-like” linkages, e.g., the apical SC membrane to the myofibre plasma membrane [150,200,201]. Together integrins, the DGC and cadherins enable the mechanical ECM–SC–myofibre linkage and external signalling necessary to facilitate mechanical tension sensing, and mediate the steps shown in Figure 5: i.e., SC activation, migration, changes in gene expression, proliferation, differentiation and fusion of the new myonuclei with the myofibre enabling muscle growth. This requires controlled SC migration under the basement membrane coupled with complex and continuous intra cellular signalling to the SC nucleus. Thus, bidirectional mechanotransduction is required: “outside-in” signalling from the extracellular matrix to the SCs to activate and transform them, and “inside-out” force transmission from SCs to the ECM and myofibre outer surface to mediate SC migration. At the same time, the ECM external environment of SCs must undergo considerable remodelling to allow the increased muscle length and CSA of myofibres, fascicules and the whole muscle, i.e., complete remodelling of the endo, peri and epimysial ECM. Whilst a large number of factors and cell types, many of which are shown in Figure 4, are known to be involved, a detailed account of this process has yet to be described. What has been described are molecular models for parts of the process and these will be briefly reviewed here.

In the case of the DGC, although its generally accepted role is to act as a molecular shock absorber in the myofibre and to stabilise the plasma membrane during muscle contraction, it is involved in outside-in signalling in SCs especially during their activation, when the DGC is important in regulating the establishment of their polarity and hence efficient asymmetric division. Thus, the DGC has been shown to mediate the epigenetic activation of Pax7 which then mediates the transcriptional activation of the myogenic determination factor Myf5 [199].

Most is known about the integrins which have the ability to bidirectionally transduce forces exerted from the extracellular matrix (ECM) into cellular biochemical signals that regulate the cell cytoskeleton of both SCs and the myofibre [202]. They are heterodimeric trans membrane proteins comprised of α and βsubunits, of which there are a large number expressed in different cell types [203,204]. Each integrin subunit includes a large extracellular domain, with specificity for a ligand, which includes ECM proteins, such as fibronectin, (integrin α5β1), laminin, (integrin α7β1) and SPARCL-1. Human SCs and the myofibre express the laminin α7β1 integrins when proliferating and differentiating [138,201,205]. The link between the extracellular head of the integrin-associated complex and the intracellular tail anchored to the actin cytoskeleton includes a molecular bridge formed by functional-multiprotein assemblies described as signalling, actin linkage and actin regulatory-modules [56,206] which together achieve mechanotransduction. Key adaptor proteins include talin, vinculin, tensin, focal adhesion kinase and paxillin, with α-actinin and zyxin important as cytoskeleton linkage proteins. Focal adhesion kinase (FAK), a large non-receptor tyrosine kinase that localises to focal adhesion complexes, represents a key component of integrin-mediated signalling which autophosphorylates in response to cell adhesions and can link to actin via paxillin and vinculin [207].

Cadherins, like integrins, are a major class of calcium-dependent adhesion proteins [208,209] which can be involved in force transmission [206,210,211]. Unlike integrins, extracellularly, they bind with cadherins on other cells and both N- and M-cadherins are expressed on muscle myofibres and SCs [139,150]. Cadherin intracellular regions bind directly to β and γ catenin, which in turn bind to α-catenin which links directly with the F-actin cytoskeleton. It also links indirectly via the same vinculin adaptor protein which acts as a mechanosensor for the integrins.

**Figure 7:**
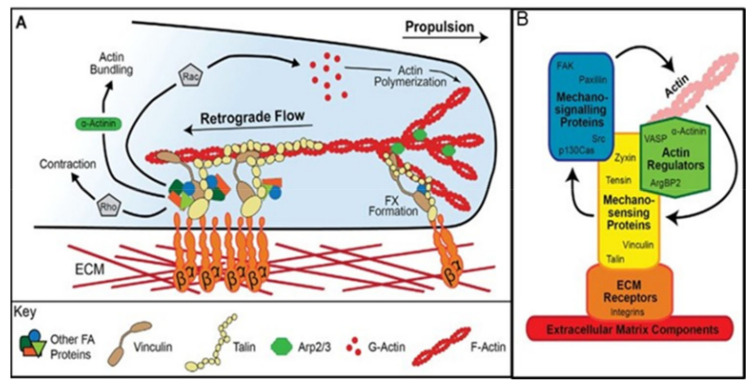
Integrin-based models of mechanotransduction which could operate in muscle satellite cells. (**A**) In the clutch model for SC migration, actin polymerisation at the advancing leading edge of the cell generates a retrograde flow of F-actin. Talin and vinculin both engage with the retrograde flow of actin, which generates tensional forces across them, regulating their activation states. Talin and vinculin bind to several signalling proteins including focal adhesion kinase, paxillin and others, producing downstream signalling events regulating the activity of the Rho-family GTPases. Vinculin can also induce actin bundling, acting through α-actinin. The activation state of talin and vinculin regulates their dynamic turnover, as well as influencing the molecular stoichiometry of the adhesion to direct downstream signalling to Rho-family GTPases. Rho activation leads to greater contractility and FA growth, whereas Rac activity enhances actin polymerisation at the leading edge. The growth of new actin filaments also leads to the formation of focal complexes (FXs), which can mature into larger focal adhesions, which act locally to control intracellular signalling. The sensing of mechanical stimulation (i.e., pulling on the integrin receptors during ECM stretching), or by the cell actively probing its ECM environment, utilises this linkage between the actin cytoskeleton and integrin-associated complexes (IACs) to transduce cellular migration. (**B**) IAC proteins can be roughly classified into the different groups shown based on their turnover. The ECM components (e.g., fibronectin) themselves are turned over, albeit slowly. The ECM receptors, such as integrins, have a slower turnover than any other IAC components. Mechanosensing proteins are those whose turnover rate has been reported to change in response to substrate stiffness. Mechanosignalling proteins do not change their turnover speed in response to substrate stiffness but are essential for generating the intracellular responses to mechanical stimuli. Finally, IACs contain proteins, the actin regulators with a slower turnover than the mechanosignalling proteins but faster than the mechanosensing proteins, which are responsible for locally regulating the actin cytoskeleton. Copied and modified from Jansen, Atherton and Ballestrem, 2017 [56] under the terms of the Creative Commons Attribution 4.0.

**Figure 8:**
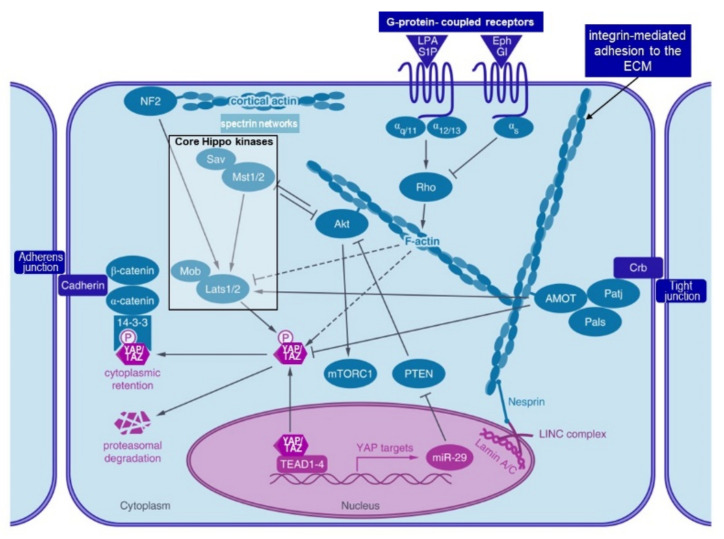
The hippo pathway and the role of YAP and TAZ in mechanotransduction of SC activity. The core components of the hippo pathway are serine/threonine kinases Mammalian Ste20-like 1/2 kinase (MST1/2) and large tumour suppressor 1/2 kinase (LATS1/2), which act to phosphorylate YAP (Yes-associated protein) and TAZ (transcriptional co-activator with PDZ binding motif), to determine the activation state of the pathway. When YAP and TAZ are unphosphorylated they are active and able to enter the nucleus and activate transcriptional enhancer factor, (TEAD)-mediated gene expression. After phosphorylation by LATS1/2 kinase, YAP binds to 14-3-3 proteins, leading to its cytoplasmic retention and also to its degradation. YAP activity is regulated by the actin cytoskeleton which inhibits YAP/TAZ phosphorylation and which is linked to the ECM via integrins. Actin stress fibres connect to the lamin meshwork in the nucleus via the LINC-complex, (Linker of Nucleoskeleton and Cytoskeleton). Rho GPTases are regulated by G-protein coupled receptor signalling which in turn regulates actin dynamics and YAP activity (dashed lines). Actin-binding proteins, like angiomotin (AMOT) or neurofibromin 2 (NF2/Merlin) are also known to regulate YAP activity, either through LATS or by direct interaction with YAP. Akt, a key regulator of the IGF-1-mTOR pathway, also binds to actin stress fibres and crosstalks to the Hippo pathway by interacting with MST1/2 and by YAP-induced expression of a microRNA (miR-29) which inhibits the inhibition of Akt by targeting the phosphatase and tensin homolog, PTEN. Copied and modified from Fischer et al. 2016 [231] with extra detail from [232].

**Figure 9:**
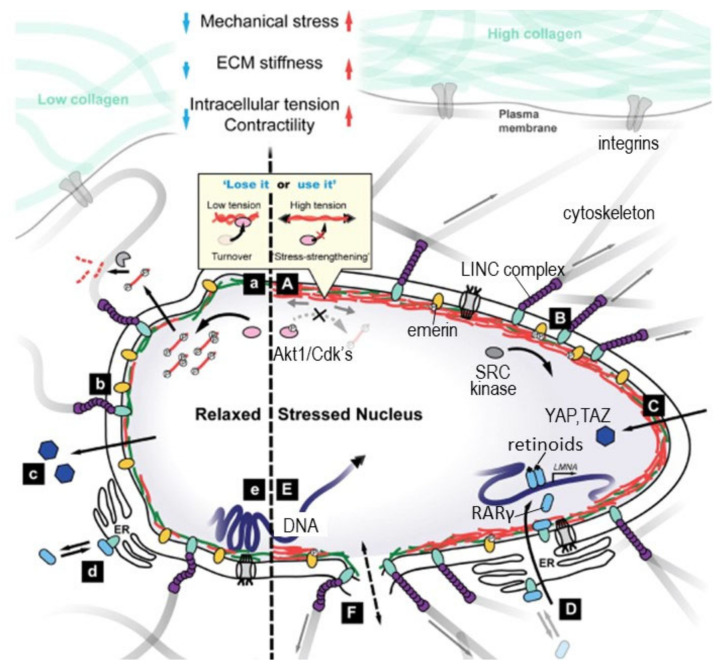
Mechanosensing by the nucleus. (A and a) High nuclear tension can induce conformational changes in lamin A, C and B1/B2 coiled-coil dimers, which sterically inhibits access by kinases, Cyclin-dependent kinases (Cdk’s), protein kinase C (PKC), and protein kinase B, (Akt), which would otherwise increase lamin turnover. (B and b) Pulling on nesprin-1, (the LINC complex of Nesprin and sun domain-containing protein 2 (SUN2) which tethers the nucleus to the cytoskeleton), leads to phosphorylation of emerin by non-receptor tyrosine kinase (SRC) with stress stiffening of the nucleus influencing downstream mechanoresponses: e.g., formation of stress fibres, migration, localisation of YAP and TAZ, and serum response factor transcription. (C and c) Mechanosensitive transcription factors such as YAP and TAZ, which influence growth in the Hippo pathway, (see Figure 8), translocate into the nucleus under stress to modulate gene expression. (D and d) Mechanical stress leads to nuclear localisation of RARγ, (retinoic acid receptor γ) which directly regulates LMNA (lamins A and C gene) transcription. (E and e) Application of mechanical force may lead to changes in chromatin conformation (e.g., local stretching of genes), thereby altering transcriptional activity. (F) High tension can induce membrane dilation and may lead to transient ruptures, allowing for the exchange and mislocalisation of nucleoplasmic and cytoplasmic factors. Copied and modified from Cho, Irianto, and Discher 2017 [212] under a Creative Commons License (Attribution–Noncommercial–Share Alike 4.0 International license).

An “outside in” model of mechanotransduction of satellite cells is also supported by the integrin, talin, vinculin focal adhesion as shown in Figure 7B [56] which could mediate signalling from the ECM induced by stretch or shear forces as shown in Figure 6, enabling a linkage to the nucleus via the actin cytoskeleton. This links to proteins within the nuclear envelope such as lamins which transmit forces into the nuclear interior, enabling regulation of gene expression by a number of mechanisms [212], (see Figure 8; Figure 9).

In an “inside-out” model of force transduction from the SF to the ECM, traction forces from SFs can be exerted through the integrin connections to the ECM, in some cases sensing the stiffness of the ECM and moving the cell towards regions of increased stiffness, “durotaxis”. Several molecular mechanisms have been proposed involving fluctuations in myosin contractility, cyclic variations in the assembly/disassembly of actin molecules in the SF, and fluctuations in the FA “molecular clutch” [195,213]. The “molecular clutch” is conceptualised as temporal variations in the strength or number of linkages between SFs and the ECM via changes in protein–protein interactions within FAs, possibly at the level of the integrin head–ECM protein linkage. A model for such a mechanism is shown in Figure 7A [56]. Cadherins like integrins can also mediate inside-out traction forces enabling SC migration along the outside of the myofibre under the BL with a molecular clutch mechanism [206]. Another function of the cadherin-based FA between SCs and the myofibre is to maintain SC quiescence in their niche, possible in conjunction with hippo signalling, (see below), to the nucleus via YAP [214], as long as the adhesion is strong. If the adhesion is disrupted the SC can become activated [200].

#### 4.3.3. Role of mTORC1

In addition to direct cytoskeleton-related nuclear activation by mechanotransduction, several additional signalling pathways appear to be involved. mTOR plays a central role [215], and the mTORC1(raptor containing) activity is high during the proliferative phase of embryonic myogenesis and low during cell fusion and fibre maturation, and while raptor knock-out embryos died after birth, [216,217], the inactivation of mTORC1 interfered with, but did not completely prevent, embryonic myoblast proliferation and differentiation [218]. In adult mice, abrogation of raptor in SCs did not alter the size of the stem cell pool under homeostatic conditions but severely impaired their capacity to regenerate skeletal muscle owing to defects in proliferation consistent with previous observations that mTOR has low activity in quiescent satellite cells but becomes active on SC activation [154]. This implies that mTORC1 is important when demands for protein synthesis are high during the proliferative phase of the myogenic sequence.

In contrast, there is little to suggest a key role for mTORC2 (Rictor containing) activity given that Rictor KO mice embryos were viable with normal overall muscle histology at young ages [218] and do not display any overt phenotype apart from some metabolic changes in muscle [219]. Thus, although mTORC2 is said to control cytoskeletal remodelling, cell metabolism and survival [220], it does not seem to play a decisive role during SC-induced myofibre hypertrophy.

Since the Akt–mTOR signalling pathway has long been known as an important component of the muscle hypertrophy response [60,221], IGF-1 was initially seen as the main activator of this pathway [222] and important in the regulation, with IL6, of excessive fibrosis during muscle repair after injury [177]. In transgenic mice, the upregulation of IGF1 expression in mouse skeletal muscle [223], dramatically elevated IGF1 levels and stimulated the IGF1/Akt signalling pathway when the mice were growing at one month. This modestly increased muscle size through increasing fibre CSA but only significantly in female mice. Circulating IGF1 levels and tibia length was not affected confirming that muscle IGF-1 is not normally a major source of circulating IGF-1. The authors speculated that the lack of an effect in the male mice may have been because the IGF-1 pathway was already maximumly stimulated. The hypertrophic effect of the increased IGF-1 observed only in the young growing animals is consistent with another transgenic study of overexpression of muscle IGF-1 in which muscle hypertrophy and increased activation of the Akt pathway is only apparent at the time of usual rapid growth in young animals or during muscle regeneration in dystrophic muscles [224] when myogenic differentiation was occurring as indicated by increased expression of myogenin, a key myogenic regulatory factor. However, muscle hypertrophy can be induced without a functioning insulin/IGF-1 receptor [225], indicating that the activation of mTOR and muscle growth can be achieved bypassing the IGF-1 receptor [226].

One potential upstream mechanotransduction activation pathway to mTORC1 and the ribosomal protein S6 kinase, S6K, is via an integrin-FAK-actin microfilament complex. This has been demonstrated in shock-wave exposed mesenchymal stem cells, resulting in their maturation and proliferation in culture [227] suggesting a similar pathway in SCs. As to the downstream pathway for the mTORC1 activation of the myogenesis programme, the Per–Arnt–Sim domain kinase (PASK), is one link [228]. PASK is a downstream phosphorylation target of mTORC1 and PASK phosphorylates Wdr5 to stimulate SCs differentiation by epigenetically activating the myogenin promoter [229]. This results in exit from SC self-renewal, and induction of the myogenesis program. Following this mTORC1-S6K signalling is required for myoblast fusion at the later stage of myogenesis [229].

#### 4.3.4. Hippo Pathway and Notch Signalling

Another important signalling mechanism is the highly conserved hippo pathway [212,230,231,232,233,234]. This is a key regulator of organ size and tissue homeostasis, connecting a number of primary signals including those acting through the actin cytoskeleton with regulation of nuclear transcription via two effectors: Yes-associated protein (YAP) and transcriptional co-activator with PDZ binding motif (TAZ). As transcriptional co-activators, YAP/TAZ cannot bind DNA directly, but interact with DNA-binding transcription factors, e.g., the TEAD family of transcription factors, to regulate target gene expression. The main features of the pathway are shown in Figure 8. Studies in mice indicate that TAZ and YAP have overlapping functions in promoting SC proliferation but TAZ then switches to enhance myogenic differentiation [231,235]. YAP signalling has been suggested to mediate integrin signalling in the myofibre during skeletal muscle hypertrophy in response to a mechanical stimulus [205]. These authors suggest a two-step process within the myofibre following mechanical loading in which mTORC1 is activated by amino acid uptake or release from the lysosome to rapidly increase protein synthesis to establish homeostasis, followed by sustained maintenance and remodelling of muscle structure via an integrin–ILK–mTORC2–YAP-driven mechanism.

The hippo pathway is also important in relation Notch signalling since crosstalk between YAP/TAZ and Notch has been identified [236,237]. Notch signalling promotes cell–cell communication through juxtacrine signalling, enabled by the interaction between single-pass transmembrane ligands of the Delta–Serrate–Lag (DSL) family on one cell and the Notch protein receptor on the other. As shown in Figure 5 quiescent SCs express high levels of the Notch receptors as well as the intracellular mediators of Notch nuclear activation, but after SC activation Notch receptors fall to low levels and stay low during SC proliferation and differentiation stages, apart from the small population which undergo self renewal and repopulate the niche [238]. It appears that the Notch ligand Jag2 within the myofibre is induced by YAP/TAZ during normal muscle contractions and this interacts with the SC Notch receptor preserving their potency and quiescence and preventing their differentiation [237].

#### 4.3.5. WNT Signalling

The Wnt signal transduction cascade is another important regulator of stem cell development [239], including SC activation [240]. Wnts are a family of secreted glycoproteins which bind to specific Frizzled (Fzd) transmembrane receptors. Syndecan-4 (Sdc4), a plasma membrane proteoglycan, is an Fzd7 coreceptor and both are expressed and required for Wnt7a signalling in SCs. Quiescent SCs express the Wnt receptor Fzd7 and on activation express its candidate ligand Wnt7a and undergo symmetric SC expansion through the planar cell polarity (PCP) pathway [241] which signals through the actin cytoskeleton [240]. This mechanism also requires activated SCs to secrete fibronectin (FN) into their niche which binds with both Wnt7a and the Fzd7/Scd4 receptor complex [242]. SC differentiation is controlled mostly through the canonical β-catenin pathway in which β- catenin accumulates and translocates into the nucleus to complex with TCF/LEF(T-cell factor/lymphoid enhancer factor) transcription factors to induce context-dependent Wnt/β-catenin target genes. Binding of Wnt to the Fzd receptor also leads to the release of intracellular Ca^2+^ mediating the calcium-dependent responses discussed below. Finally, Wnt7a expression/application leads to hypertrophy in resting adult skeletal muscle and in muscle cell culture by activating PI3K and the AKT/mTOR anabolic pathway independently of IGF receptor activity. However, it is not clear whether canonical Wnt signalling, (e.g., through Wnt4), is indeed inducing hypertrophy or if increased myotube diameters are due to accelerated SC differentiation associated with Wnt7a expression (see Figure 5). In developing mammalian tissues, mammalian embryo Wnt promotes morphogenesis in part by inducing expression of the system L1 amino acid transporter isoform Slc7a5 which delivers leucine and other large neutral amino acids (LNAAs) to support metabolic demand for amino acids [243], and this may also occur in SCs.

#### 4.3.6. Free Calcium

Changes in free Ca^2+^ in response to stretch-induced Ca^2+^ influx into satellite cells is another important mechanism for regulating gene expression [244,245,246]. Mechano-gated Ca^2+^ channels, especially transient receptor potential (TRP) channels, can increase intracellular concentrations initiating calcium-dependent responses. One pathway involves calcium binding to calmodulin and then activating calcineurin, the calcium- and calmodulin-dependent serine/threonine protein phosphatase which dephosphorylates nuclear factor of activated T cells (NFAT). This then translocates into the nucleus to initiate myogenic gene transcription (including MEF2, MyoD, Myf5 and myogenin). In addition, increased calcium can effect signalling from extracellular ligands including FAK through several elements of the mitogen-activated protein kinase (MAPK) pathway and this regulates different steps of myogenesis [247,248] including the cyclin D1-mediated increase in cell proliferation [246].

A summary of many of the above mechanisms relating to mechanosensing by the nucleus is shown below in Figure 9. 

#### 4.3.7. Metalloproteinases

The ECM remodelling, which is a central feature of SC-induced muscle growth, is enabled by matrix metalloproteinases (MMPs). These are a large family of calcium-dependent zinc-containing endopeptidases, secreted by SCs and several other muscle cell types including macrophages [26,249] which can degrade collagens, elastin, fibronectin, laminin, and proteoglycans within the ECM. For example, activated SCs upregulate MMP2 and MMP9 expression, leading to a local digestion of the laminin-α2-containing basal lamina at the SC niche. Additionally, SCs provide an essential autocrine source of MMP-13, which regulates their migration beneath the basal lamina [250]

.

From the above, it is clear that mechanisms exist to mediate the mechanotransduction of postnatal SC-induced muscle growth by passive stretch of muscles subsequent to bone length growth. These are coupled with crosstalk via cytokines and growth factors, and the cells shown in Figure 4. Together these factors mediate both myofibre hypertrophy and the reengineering of the ECM. To what extent the events in children and adolescents differ from those observed in the experimental systems from which the above mechanisms have been distilled, remains to be clarified

### 4.4. Myofibre Hypertrophy and Its Implications for Protein Turnover within the Myonuclear Domain

**Figure 10:**
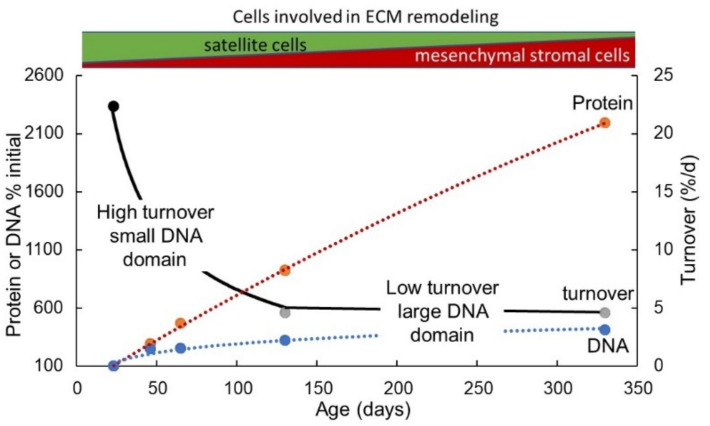
Myofibre growth and protein turnover in rat muscle. At weaning, the protein:DNA ratio is small partly because of a high proportion of satellite cells but also because within the myofibre the DNA domain size is small because each nucleus has to manage protein with a high turnover rate. The fall in the turnover rate with age allows each myonucleus to manage a larger domain allowing rapid growth of myofibre protein. The turnover rate shown is the fractional breakdown rate calculated as the measured in vivo rate of protein synthesis shown in well-fed rats in Table 2 minus the muscle growth rate [255].

In 1973, we introduced the concept that identified the dietary protein-induced regulation of muscle growth acting via stimulation of both the capacity for and efficiency of ribosomal mediated protein synthesis: i.e., total RNA concentration and protein synthesis per unit RNA [251]. Thus, new myonuclei can increase protein synthetic capacity in terms of ribosomal RNA, [58,252] enabling increased global myofibre protein synthesis [60]. Because each myofibre is a multinucleated, post-mitotic single cell, each myonucleus is envisaged as controlling a volume or domain of the fibre, originally defined in operational terms as a DNA-unit with the dimensions of the protein-DNA ratio [253]. Total muscle DNA was used as a proxy for the number of diploid myonuclei, on the basis that the proportion of myonuclei to total nuclei present did not change with age in rat skeletal muscle [254] although there was no certainty in that assumption. In both animal [255] and human muscle [129], postnatal growth involves increases in both the number and size of myonuclear domains. In human muscle, growth from birth to puberty was shown to involve a sixfold increase in the myofibre CSA, a 2–3-fold increase in numbers of myonuclei per fibre, an increase in the myonuclear domain which is rapid in infants and much slower during school age, and a steep initial decline in SC density followed by a low plateau [129]. The implication of such growth for the myonuclear direction of protein turnover in its domain was revealed to some extent with measurement of DNA and RNA content, domain size and in vivo rates of protein synthesis, the fractional synthesis rate, (FSR), as %.day^−1^, in two of the leg muscles from colonies of well-fed and marginally protein-deficient rats [256] over 330 days of growth [253,255]. This allowed the DNA activity, g protein synthesis.d^−1^gDNA^−1^ to be quantified as a function of both ribosomal content, RNA/DNA, and efficiency, g protein synthesis.d^−1^gRNA^−1^ a measure of the rate of translation per ribosome. As shown in Table 2A, in early postnatal life in immature rapidly growing rat muscle there is a high FSR (%.day^−1^), indicating a high protein turnover rate, i.e., extensive proteolysis and replacement of myofibre proteins associated with the structural remodelling of the newly formed myofibres. This is achieved within a small myonuclear domain within which its ribosomal complement (RNA/DNA and RNA/protein ratios) achieved higher rates of protein synthesis than at later stages of muscle development. Subsequent enlargement of the domain is achieved in association with falling rates of protein turnover with little change in the ribosomal complement but with declining rates of RNA activity. At 1 year, the domain size had increased by a factor of >5 times with the protein turnover rate proportionately lower (<20%) and managed by an increased ribosomal complement with similar values of RNA activities compared with all but the most immature muscles. Importantly overall rates of protein synthesis per domain did not change much after 6 weeks of age. Thus, the key changes which allowed expansion of the domain was the decreasing protein turnover rate. This is shown graphically in Figure 10.

Marginal protein deficiency slowed growth and reduced muscle mass by about 50% in the fully mature animals at 330 days. The dietary effect was most marked in the youngest animals who showed a marked sensitivity to the marginal protein deficiency, with fewer myonuclei, a smaller myonuclear domain size and with protein synthesis per domain reduced to only 25% compared with the well-fed animals. This was because of lower turnover rates, a lower ribosomal RNA capacity and lower RNA activity. From 60 days, protein synthesis per domain and ribosomal capacity and activity recovered to control or near control levels but a 50% size deficit remained at 330 days through 33% fewer myonuclei, a 20% smaller myonuclear domain with 30% lower rates of protein synthesis per domain, a clear example of the nutritional sensitivity of muscle growth.

The inverse relationship between protein turnover rate and domain size also obtains for adult muscles with different fibre types associated with different structures, functions and protein turnover rates. This is exemplified by the avian posterior and anterior latissimus dorsi muscles (PLD and ALD). The PLD contains principally fast twitch fibres with the ALD muscle characterised by slow tonic fibres. Myonuclear domain sizes are quite different (PLD = 2.4 × ALD) with size inversely related to protein turnover rates (PLD = 40% ALD) but with similar DNA activity [257], and in this case it is unlikely that the differences in DNA concentration reflect different number of non-muscle myonuclei. Finally, in response to the stretch-induced hypertrophy of the ALD, characterized by immediate dramatic increases in muscle nuclei and non-collagen protein synthesis, (as well as collagen synthesis [61] not shown), which outstrips initial increases in weight and protein, the eventual >100% increase in size over 1–2 months was accompanied by similar increases in protein and DNA, maintaining the same domain size, turnover rate and DNA activity [59].

These findings suggest that the turnover rate and domain size are a physiological feature of muscle function and structure: i.e., the more ordered linear structure of sarcomeres in fast twitch muscles result in a slower rate of turnover than the highly branched structure in slow tonic muscles which exposes a larger proportion of myofibrillar proteins to proteolysis. This requires in turn higher rates of replacement protein synthesis only achievable by higher myonuclear concentrations-smaller domain sizes.

### 4.5. Implications of Changes in Total Muscle Nuclear Domain Number and Size for ECM Remodelling during Postnatal Growth

The compositional changes during postnatal growth shown in Table 2 raises an important question in relation to the cellular mechanisms of postnatal bag enlargement. Rats become sexually mature at about 50 days of age but the male displays continual growth in length and weight throughout adulthood because the growth plate does not fuse (as it does in the female rat). As shown in Table 2, there is a more than fivefold increase in muscle mass post-puberty (at 50 days) and this must involve the mechanotransduction of ECM remodelling allowing “bag enlargement” through activation of both SCs and associated mesenchymal stromal cells (see discussion above in Section 2 in association with Figure 4). Growth in total protein outstrips total DNA, as shown in Table 2 and Figure 10, indicating an expansion of domain size possible because of a fall in turnover rate. This reduces the requirement for new protein synthetic capacity from new myonuclei from activated SCs, consistent with the relatively modest increase in total DNA. This makes it less likely that activated SCs can achieve the requisite ECM remodelling required for the marked myofibre hypertrophy. This would result in an increasing dependency on activation of existing mesenchymal stromal cells (shown in Figure 4) to achieve the mechanotransduction of the necessary bag enlargement within the ECM and this would be less noticeable in terms of an increase in total cell number and DNA. Clearly the composition of the muscle cells contributing to ECM synthesis and to the increase in total DNA during growth and development is an important question to resolve.

## 5. Regulation of Protein Deposition in Muscle: Experimental Studies in Animals and Human Adults

Clearly after the initiation of SC activation, insertion of new myonuclei into the myofibre and remodelling of the ECM allowing “bag expansion” as described above, the growth of the musculature will require sufficient protein, associated nutrients and the associated endocrine anabolic drive to mediate “bag filling” in terms of net muscle protein synthesis. A central feature of the Protein-Stat is that this provision is assured by an appetite mechanism which links the metabolic demand for protein for muscle growth to appetite through an amino-static mechanism (see Millward 1995 for details [7]).

### 5.1. Animal Studies of Regulation of Protein Deposition in Muscle: Dietary Protein, Insulin, Amino Acids and Thyroid Hormones

Although it has been suggested [258] that the signalling modules responsible for skeletal muscle growth during development include not only positive nutritional and endocrine regulation via the IGF1–PI3K–Akt/PKB–mTOR pathway but also the negative regulation by the myostatin–Smad3 pathway which must be blocked by follistatin to allow anabolism, a role for myostatin in muscle growth appears to be limited to the foetal phase of hyperplasia, i.e., myofibre formation from myotubes [259], and regenerating muscle after injury [144]. Because this review is concerned with the nutritional regulation of postnatal muscle growth, myostatin will not be discussed further here.

The primary anabolic regulatory role of dietary protein for neonatal muscle growth was established in animal studies over many years, its target being protein synthesis (MPS), rather than proteolysis, (muscle protein breakdown, MPB) [260]. As shown in Figure 10, MPB is high in muscle during rapid growth and falls during development [255,261] and with growth suppression apart from after extended periods of food deprivation when MPB is elevated [260] in association with increased glucocorticoids [262]. Changes in both ribosomal capacity and efficiency (e.g., RNA/protein and protein synthesis/RNA as in Table 2) are both quantitatively important in mediating both growth-related stimulation and protein-deficiency induced reductions in MPS over time [251,263,264] with ribosomal efficiency responding rapidly, within 20 min of feeding [262].

In early postnatal life, protein-containing food stimulates both insulin and amino acid levels [263], and each of these responses stimulates MPS [261]. The role of insulin was established in early studies [265,266,267], with the feeding-induced increase in MPS blocked with anti-insulin serum [262]. Given the difficulty in establishing a role for insulin in the regulation of MPS in adult human muscle [268,269], and given the developmental changes in its effectiveness in stimulating porcine MPS it has been suggested that the ability of insulin to increase protein synthesis in skeletal muscle appears to be unique to the immature muscle [261]. However, it would appear that there may be species differences in insulin’s regulatory role since subsequent studies have clearly showed that insulin is required for the regulation of rat muscle protein synthesis irrespective of age [270].

The interaction of insulin and amino acids and the extent of their independent influence on MPS in mediating the feeding response initially proved difficult to establish, not least because many studies showed that administration of insulin without amino acids lowers amino acid levels, and administration of amino acids or leucine alone increases insulin levels. In studies where they were both controlled independently it was demonstrated that insulin was permissive for the maximum influence of amino acids, especially leucine, [265,267,271,272] and eventually established that insulin at low physiological levels and amino acids act through independent upstream pathways to activate mTORC1 stimulation of protein synthesis. Thus, insulin signals to mTORC1 through an Akt/Rheb pathway, whereas leucine signals through the Rag pathway, interacting via the cytosolic sensors sestrins [60,125,261,273], probably sestrin1 [274] (see below for a more extensive discussion).

**Figure 11:**
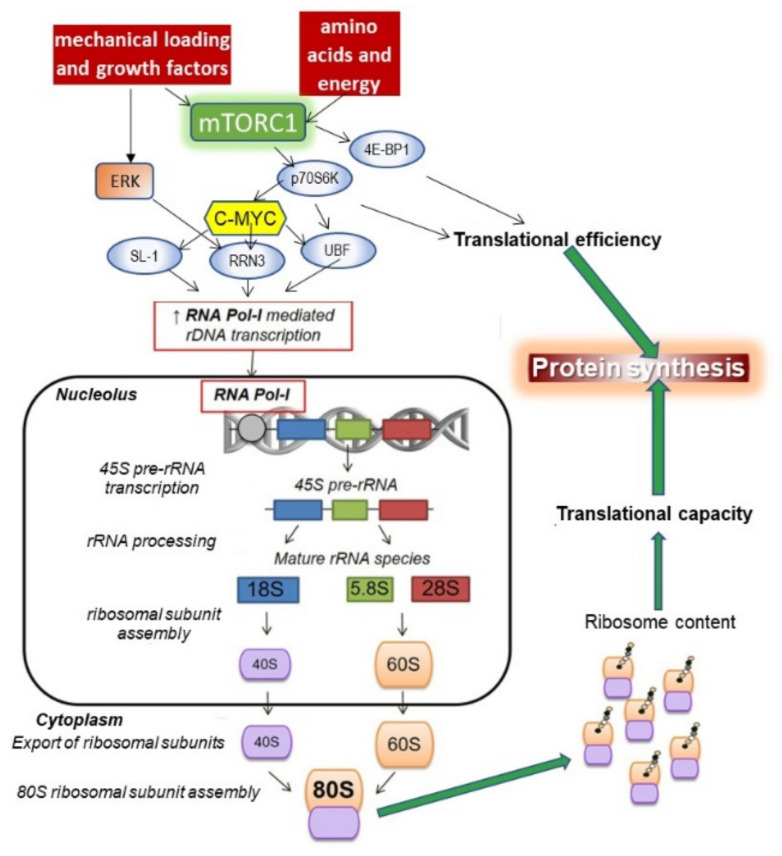
Regulation of ribosomal capacity and efficiency of muscle protein synthesis by nutritional and mechanical factors. Multiple signalling pathways including extracellular signal-regulated kinase (ERK) and mTORC1 mediate activation of the nucleolar RNA Polymerase I (Pol-I) by a number of factors which form a transcriptional complex, the “Pol I regulon”, at the rDNA promoter [275]. The elevated expression of the proto-oncogene transcription factor C-MYC, achieved through mTORC1/S6K1 [287], plays a central role stimulating the Pol II-dependent transcription of a cohort of factors associated with Pol I regulon including RNA polymerase I-specific transcription initiation factor RRN3, (also known as TIF-1A, transcription initiation factor 1A) and upstream binding factor (UBF). Ribosome biogenesis involves transcription of the 45S ribosomal RNA precursor (45S pre-rRNA), processing of the 45S pre-rRNA into the smaller rRNAs (18S, 5.8S and 28S rRNAs) followed by assembly of these rRNAs and other ribosomal proteins into ribosomal subunits (40S and 60S) which are exported into the cytoplasm. The 80S ribosome is the mRNA translating unit, requiring mTORC1-mediated activation of initiation through p70S6K (p70 kDa ribosomal protein subunit kinase 1) and 4E-BP1 (eukaryotic initiation factor 4E binding protein 1). mTORC1 also regulates RNA Pol-II (which transcribes the ribosomal proteins and other proteins required for transcription) and Pol–III, (which synthesises small structural RNAs such as 5S rRNA and transfer RNA (tRNA) (not shown). Modified from Fyfe et al. 2018 [288] under a Creative Commons Attribution 4.0 International License.

The dietary regulation of muscle ribosome levels has been much less investigated. Ribosome biogenesis is a complicated and energetically costly process requiring production of four ribosomal RNAs (rRNAs) and the synthesis of ≈80 ribosomal proteins that make up the mature 80S eukaryotic ribosome. This requires activation of three classes of RNA polymerases (POL I, II, and III), with synthesis of precursor rRNA (i.e., 45S pre-rRNA), by POL I the a major rate-limiting step and its regulation involving multiple signalling pathways [252,275,276] including ERK, AMPK, mTORC1 and P70S6K1. This allows for ribosomal biogenesis to be controlled by hormones, nutrients, including amino acids [277] and contractile activity. In addition, the Wnt/b-catenin/c-myc mechanically sensitive signalling pathway involved in regulating cell growth is involved in virtually all aspects of ribosome formation and is highly expressed during skeletal muscle hypertrophy [276]. Insulin may well be one important control factor since it has been shown to regulate ribosome content in primary cultures of rat hepatocytes by accelerating the rate of transcription of rDNA and by slowing the rate of ribosome degradation which occurs after polyribosomal disaggregation when mRNA translation is inhibited [278]. In fibroblasts and hepatoma cells, insulin activates POL I [279]. Some of these pathways are shown in Figure 11.

Thyroid hormone status, which varies in the rat with dietary protein levels [264], also appears to be a controlling influence on ribosomal capacity in muscle as indicated by treatment of thyroidectomised rats with either T4 [280] or the active hormone T3 [281,282,283]. How T3 regulates ribosomal RNA levels is uncertain. The major effects of thyroid hormones are mediated by modulation of gene transcription through thyroid hormone receptors acting on thyroid response elements in target genes. T3 treatment in healthy men upregulated 257 identifiable genes of which the largest fraction was involved in the transcriptional and post-transcriptional control of protein synthesis, including ribosomal proteins and translation initiation factors, ribonucleoproteins and RNA metabolism, signal transduction and proteolysis by the ubiquitin/proteasome pathway [284]. Cytosol-localised TRα1 receptor may also be involved since in cultured cardiomyocytes T3 can act through this receptor to activate the PI3K–Akt signalling pathway allowing physiologic cardiac growth [285]. Whilst in children with severe malnutrition thyroid hormone status is reduced (possibly as an adaptive response [286]), as it is in areas of iodine insufficiency where it can be associated with stunting ([120]), there is little evidence that thyroid hormone status reflects protein intake in otherwise healthy children.

### 5.2. Animal Studies of Muscle and Bone Growth Interactions

**Figure 12:**
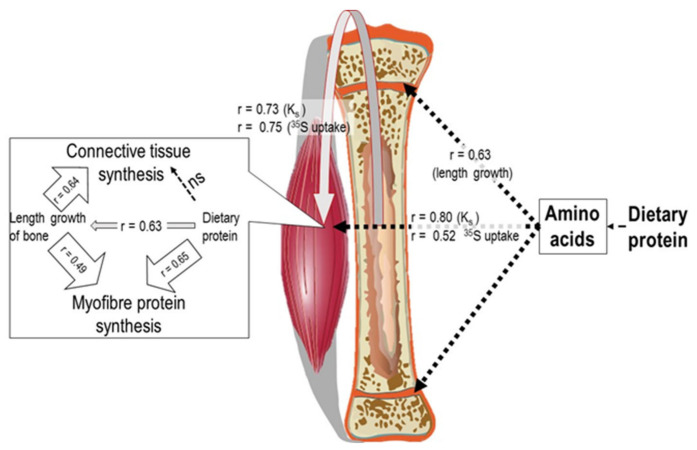
Influences of dietary protein on muscle and bone growth and their interactions [291]. Relationships indicated by full and partial correlation analysis of the interaction between dietary protein intake and bone length growth as determinants of muscle myofibre protein synthesis (Ks) and muscle connective tissue synthesis (^35^S uptake) in the young rat measured during the first 3 days of variable dietary protein intakes (20%, 7%, 3.5% and 0.5% dietary protein). Bone length growth, muscle Ks and ^35^S-uptake were each significantly correlated with dietary protein intake and muscle Ks and ^35^S-uptake were each correlated with bone length growth. The independent influences of bone length growth and dietary protein on muscle is indicated by the partial r values in the box on the left, showing that the apparent influence of protein intake on ^35^S uptake in muscle is entirely accounted for by its influence on bone length growth, which in turn influences muscle ^35^S uptake and myofibre Ks to a lesser extent. Bone length growth was indicated by the more sensitive index of epiphysial cartilage width which is proportional to and an early indicator of, changes in overall tibial length [289].

The contribution of bone length growth-induced muscle stretch as an additional indirect fluence of dietary protein on muscle growth, whilst an obvious part of the coordination of muscle and bone growth, has been poorly investigated. Protein deficiency and bone growth was investigated in animal studies in the 1950s and 60s (see [289] for a summary of early work), and in relation to stunting in children [120], but bone–muscle interactions had been generally ignored. However, some years ago in a comprehensive series of dietary studies, the responses of protein and proteoglycan synthesis in the tibia and gastrocnemius muscle to changes in their growth in response to dietary protein and energy restriction was described in detail [289,290,291,292,293]. An obvious direct influence of dietary protein on the length growth of the tibia was indicated, which was more profound than that of dietary energy, so that dietary protein’s anabolic influence on bone length and muscle mass could still be observed in negative energy balance with restricted amounts of a high protein diet. The differences in the time course and extent of changes in muscle and bone growth and associated protein and proteoglycan synthesis assessed in vivo, allowed the interaction between tibial length growth and dietary protein on muscle growth to be revealed by partial correlation analysis. Length growth of the tibia (sensitively indicated by changes in epiphyseal cartilage width), and muscle myofibre protein synthesis were both influenced directly by dietary protein. In contrast, muscle connective tissue synthesis was only indirectly influenced by dietary protein via the dietary protein-induced changes in tibial length growth indicating activated muscle connective tissue synthesis through mechanotransduction mechanisms subsequent to passive stretch (see Figure 12).

### 5.3. Regulation of “Bag-Filling” and the “Bag-Full” Signal in Human Muscle

If the bag theory described above is correct, it leads to the important prediction that at maintenance, there would be a measurable limitation of the extent of postprandial protein deposition in muscle through a “bag full” signal. In other words, the homeostatic mechanisms governing the nutritional maintenance of muscle mass should include the effect of the myofibre volume-limitation by the muscle ECM which in turn should be measurable as an inhibitory signal limiting postprandial protein deposition in muscle during postprandial repletion of the postabsorptive protein losses.

Whilst most of our detailed understanding of the molecular mechanisms of dietary control of muscle protein deposition derives from animal and cell studies, animal models of muscle growth have generally been uninformative in terms of characterising mechanisms of muscle growth within the “bag filling-bag-full” model. This is most likely because in most cases, human, compared with animal muscle growth, is uniquely slow so that “bag filling” within the diurnal cycle of postprandial gains and post absorptive losses, can be considered separately from “bag enlargement”. In most rodents, especially growing rats and neonatal pigs in which muscle growth has been extensively investigated, it is unlikely that the “bag full” situation could be recognised, and this appears to be what is observed. Thus, in neonatal pigs infused with a complete amino acid mixture, increased rates of MPS are maintained for 24 h [294]. In meal-fed rats, although there is a decline in MPS 3h post-meal, it is not observed if muscle energy status is maintained by oral energy from carbohydrates or large additional amounts of leucine [295,296]. In the immediate postnatal period in human infants when there is very frequent feeding and likely to be considerable remodelling of the ECM associated with the maturation of immature myofibres, as judged by the high concentration of satellite cells which decline as growth slows [129], it may be that that the phenomenon of “bag filling” and the “bag full” signal may not be relevant. However, at an early stage during infant development they do experience periods of maintenance: one-year-olds may sleep for 10–12 h. As such, a diurnal cycle of feeding and fasting is highly likely to occur between growth periods especially if growth is saltatory as discussed above. This means that the concepts of “bag filling, enlargement and full” do become relevant to muscle growth in young children and adolescents. However, because of the obvious constraints of carrying out invasive investigations in children, most of the evidence discussed here derives from muscle studies in adults [269].

The nutritional signalling within muscle during the diurnal feeding-fasting cycle is widely discussed in terms of nutrient–hormone-driven pathways (amino acids and insulin), but almost certainly involves a less understood cell swelling-related pathway which in each case ultimately controls protein synthesis and proteolysis. This means that whilst downstream signalling from these two initiating influences must converge to mediate the changes in MPS and MPB which mediate protein deposition in the myofibre, upstream initiating signalling may differ. Here, the current understanding of amino acid- and insulin-related signalling will be briefly reviewed followed by cell swelling and evidence for and likely explanations of the “bag-full” phenomenon.

The diurnal fasting-feeding cycle of losses and replacement of muscle protein starts with a hypo-insulin and hypo-amino-acidemia postabsorptive state inducing net muscle proteolysis with efflux from muscle of glutamine, alanine and other amino acids which is reversed by the feeding-induced insulin- and amino acid-driven net protein deposition, subsequent to increased MPS and decreased MPB. As recently reviewed [268,269,297,298,299] in human adult muscle, the consensus view is that within muscle it is mediated by amino acids, of which leucine in particular does appear to be uniquely important [300,301], with no apparent influence of insulin [297,302,303], at least within the physiological range: (supraphysiological insulin levels do seem to increase MPS [304]). The apparent lack of any stimulation of MPS by insulin is in contrast to its stimulation of signalling associated with increased protein synthesis (phosphorylation of proteins in the PKB–mTOR–p70S6k pathway) [303], which is not explained. The postprandial response also includes an inhibition of MPB mediated by insulin, with no obvious role for amino acids [303,305]. However, as discussed by Atherton, Wilkinson and Smith [268], insulin is important in mediating postprandial increases in microvascular recruitment and overall blood flow into muscle, probably through nitric-oxide (NO)-dependent vasodilation of precapillary arterioles, which will increase amino acid supply to muscle and therefore contribute to the postprandial increase in MPS in this way.

#### 5.3.1. Amino Acids and MPS

**Figure 13:**
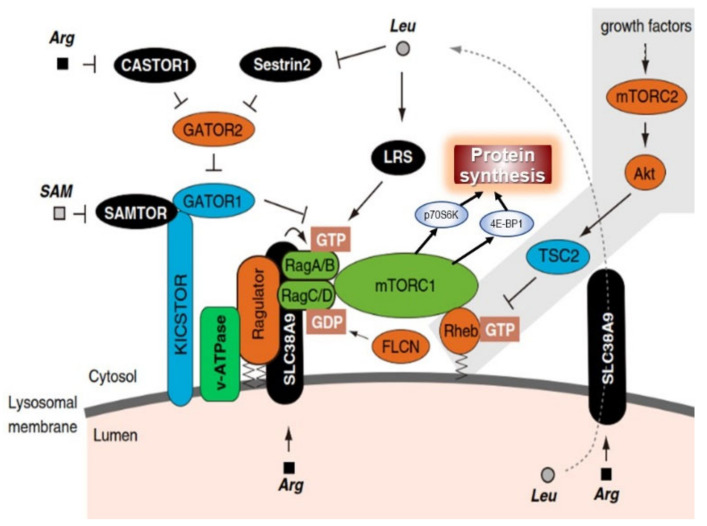
Consensus model of amino acid-dependent mTORC1 activation at the lysosome [306]. Leucine (Leu), arginine (Arg), and S-adenosylmethionine (SAM) are sensed by cytosolic and lysosomal sensors, Sestrin2, CASTOR1, and SAMTOR, (S-adenosylmethionine sensor upstream of mTORC1), as well as LRS, (leucyl-tRNA synthetase 1) and SLC38A9, (essential amino acids transporter). These signals relieve inhibitory signalling and converge on Rag GTPases (heterodimeric RagA/B-RagC/D GTPases, members of a subfamily of the Ras-like small GTPase superfamily), which recruit mTORC1 onto the lysosomal surface. There mTORC1 is activated by association with the Rheb, (Ras homolog enriched in brain), GTPase. The efflux of leucine from the lysosomal lumen to the cytosol through the arginine-sensitive SLC38A9 transporter also activates mTORC1 via cytosolic sensors. Activated mTORC1 signals an increase in protein synthesis through p70S6K and 4E-BP1 (see [308] for more details of translational control). Copied and adapted from Takahara et al., 2020 [306] under the terms of the Creative Commons Attribution 4.0.

Recent studies of amino acid-sensing mechanisms in a variety of cell types have revealed a complex system, identifying mTORC1 activation at the lysosomal surface by leucine, arginine and S-adenosylmethionine which are sensed by cytosolic and lysosomal sensors [125,273,306] (see Figure 13). Other recent studies in both murine embryonic fibroblasts and human embryonic kidney cells have indicated that a total of 10 amino acids (alanine, arginine, asparagine, glutamine, histidine, leucine, methionine, serine, threonine, and valine) were able to promote mTORC1 activity, although the time course of mTORC1 activation by individual amino acids differed considerably. Whilst they mostly signalled through Rag GTPases-dependent pathways, glutamine and asparagine activated mTORC1 through an ADP-ribosylation factor 1 (Arf1) GTPase-dependent manner [307].

In addition to these mechanisms, direct signalling to mTORC1 may occur from amino acid transporters such as the Na^+^-coupled system A/SNAT2 (SLC38A2) transporter acting as a “transceptor” which can initiate signal transduction [309,310,311]. SLC38A2 is important in maintaining the high intracellular glutamine concentration necessary to enable leucine transport into muscle by the system L (SLC7A5) transporter via exchange with glutamine.

The extent to which these mechanisms represent the events in human muscle is not known with any certainty but there is evidence that the amino acid sensing pathways in human muscle are linked to mTORC1 translocation to the lysosome as in other cell types. Thus, the expression of a number, (*n* = 22 in total) of amino acid sensing, transport, and mTORC1 regulatory genes is responsive to an increase in amino acid availability in muscle biopsies from adults 1 h after an oral dose of 10 g of essential amino acids, and this pattern of gene expression following the amino acid meal was altered following the acute disruption of lysosomal function by prior ingestion of chloroquine [312].

#### 5.3.2. Insulin and MPB

Whilst it is clear that in human adult muscle, insulin suppresses muscle protein breakdown [302,303], with amino acid supply having little influence, in contrast to many preclinical models [268], the mechanism of this action, and indeed the mechanisms of proteolysis in muscle, remain poorly understood. Any insulin-regulated proteolytic system in muscle must be able to achieve the differential turnover of individual myofibrillar and sarcoplasmic proteins described many years ago [313]. Although Fred Goldberg’s lab have presented a molecular model for myofibrillar protein breakdown by the ubiquitin-proteosome system (UPS) based on the sequential removal of sarcomeric proteins by specific ubiquitin ligases (muscle-specific RING-finger 1-MURF1, and ubiquitin tripartate motif-containing protein 32-TRIM32), with subsequent proteolysis by the 26S proteasome [314], the evidence base for this to date is limited. Others have reported evidence for removal of sarcomeric proteins by caspase 3 [315], or by calcium-dependent calpain proteinases [316] with subsequent proteolysis by the UPS or by autophagy [317,318,319,320,321,322]. It is the case that the UPS system has emerged as being highly regulated by phosphorylation [323] and to be controlled by mTORC1 [125,324,325] so that an insulin-mediated mechanism of control of muscle proteolysis by the UBS and autophagy working together as described by Zhao and Goldberg [326] is feasible. However, to date, attempts to identify the details of such an insulin-mediated mechanism in human muscle have not been entirely successful [303].

#### 5.3.3. Muscle Volume Changes and Regulation

The “Bag-theory” of muscle growth regulation [7], assumed that anabolic/catabolic signals associated with changes in muscle cell volume would be important for the “bag-filling-bag-full” signalling concept [327]. The myofibre sarcolemma, like all cell membranes, is permeable to water so that osmotically driven volume changes due to uptake or loss of water can occur rapidly. However, regulatory volume changes occur to maintain cell volume involving activation of the transport of inorganic ions, including K^+^, Na^+^, Cl, HCO^3–^ and organic osmolytes, by membrane transporters. Thus, the Na^+^-K^+^-ATPase mediates uptake of K^+^ with a net efflux of water whilst the Na^+^-K^+^-2Cl^−^ cotransporter (NKCC) mediates uptake of K^+^ and a net influx of wate [328,329], although cell volume is not perfectly regulated at a fixed volume so that moderate volume changes do occur. These are well-tolerated, but when they occur, volume changes influence the cell [327]. In hepatocytes, cell swelling acts like a pleiotypic anabolic signal with cell shrinkage being catabolic [330], an effect which Rennie had also observed with muscle [331,332] and which has been proposed to be of widespread importance to the organism [327]. It does seem to be a widespread cellular response, as indicated by the response of whole-body leucine kinetics (reduced leucine oxidation and reduced proteolysis) to the increases in the whole-body intracellular volume following a potent antidiuretic, liberal water drinking and infusion of hypotonic saline [333].

After a meal, the increases in plasma amino acid concentration accompanies an increase in plasma osmolality and Na^+^ concentration indicating water uptake into cells [334], in association with amino acid uptake to maintain the marked concentration gradient for almost all amino acids between the EC and IC space. Glutamine, in particular, is maintained at very high concentrations in muscle (≥20 mM) by the Na^+^-coupled system A/SNAT2 (SLC38A2) transporter. The large glutamine gradient allows inward transport of other neutral amino acids like leucine by the system L (SLC7A5) transporter via exchange with glutamine. Thus, in the post absorptive state, prior to the commencement of feeding, the loss of muscle protein and an efflux of amino acids would have resulted in shrinkage of the myofibre. This would allow uptake of amino acids and water with feeding allowing the sarcolemma to swell within the ECM basal lamina, activating an anabolic response with net protein deposition.

Studies in both rat liver [335] and muscle [331,336] have identified the integrin system as one major sensor of cell swelling. In muscle cells, swelling or shrinkage by exposure to hypo or hypertonic media increases or decreases glycogen synthesis and glutamine uptake, responses which are blocked by either inhibition of phosphatidylinositol 3-kinase with wortmannin or by disruption of the ECM–integrin–cytoskeleton axis (by either an integrin inhibitory peptide or disruption of microtubular or microfilamentous elements of the cytoskeleton). How the integrin system signals to protein synthesis or proteolysis has not been identified but the actin cytoskeleton can signal to mTORC1 via AKT as shown in Figure 8.

**Figure 14:**
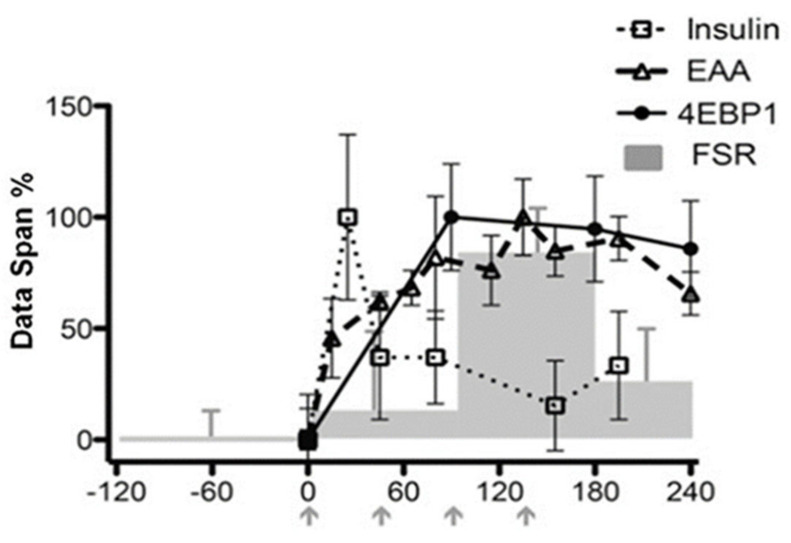
“Bag-full” effect in human skeletal muscle observed in the response of muscle protein synthesis to amino acid feeding [340]. Changes in muscle protein synthesis (FSR) measured in muscle biopsies at −2, 0, 1.5, 3 and 4 h, plasma essential amino acids (EAA), insulin concentrations and phosphor-4EBP1Thr65/70 (eukaryotic translation initiation factor 4E-binding protein 1) from fasted to fed normalised to their own data spans shown on the same axis in young men after consumption of 4 meals of 3.75 g of essential amino acids every 45 min as indicated by the grey arrows. Values are means ± SEMs, *n* = 8. The FSR was increased between 90 and 180 min (*p* < 0.05) but returned to baseline after this even though plasma EAA concentrations and muscle 4EBP1 phosphorylation remained elevated. Similar findings were reported by Atherton et al., 2010 [338] with an initial stimulation of the FSR 100 min after a single 48 g whey-protein bolus coinciding with an increase in intramuscular leucine which remained elevated at 200 min at which time the FSR had returned to baseline. Copied and adapted from Mitchell et al., 2015 [340] under the terms of the Creative Commons Attribution 3.0.

The “bag full” signal would coincide with maximal myofibre swelling when muscle amino acids, protein, water and probably glycogen content accumulate to the maximum level allowed by the muscle sarcolemma becoming in close contact with the ECM basal lamina. This is assumed to have minimal elasticity and would limit maximal myofibre volume, so that osmotic pressure between the muscle intracellular and extracellular space is equalised preventing further inward transport of amino acids and water, terminating the anabolic signal. This “bag-full” phenomenon has been demonstrated with muscle biopsy studies of MPS in adults [337,338,339,340], (see Figure 14).

Linkage between cell volume changes and mTOR have been described in relation to two different anion channels. The first, SWELL1 (LRRC8A), is a component of the volume-regulated anion channel (VRAC), which is activated by cell swelling. It has been shown to participate in the regulation of myogenic differentiation and insulin–PI3K–AKT–AS160, ERK1/2, and mTOR signalling in myotubes via GRB2-mediated signalling and to be required for maintaining normal exercise capacity and muscle endurance [341]. It has yet to be investigated whether SWELL1 participates in the post-prandial muscle cell volume increase-associated anabolic response and its termination with the bag-full state.

The second ion channel is the Na/K/2Cl cotransporter NKCC1 which is activated by cell shrinkage. This is strongly expressed in muscle and is best understood in relation to regulation of muscle K^+^ concentration in exercise [328]. However, Demian et al. [342] have reported that in HeLa and HEK293T cells, it forms a complex with and inhibits the leucine transporter LAT1 (SLC7A5) and the glutamine transporter SNAT2 (SLC38A2), and also inhibits the insulin receptor and AKT and ERK signalling, thus suppressing mTORC1 activation. Physiologically, NKCC1 would act to increase cell volume in response to cell shrinkage and such activation might account for the inhibition of glutamine uptake into muscle exposed to a hypertonic medium as reported by Low and Taylor [336], although as described above that response was shown to involve an integrin-dependent mechanochemical signalling mechanism. In muscle in vivo during the fast-feed cycle, NKCC1 could be activated in the postabsorptive catabolic phase when myofibre shrinkage occurs and could mediate part of the fasting-induced catabolic responses. However, it would be expected to be inactivated by the postprandial myofibre swelling and would only become relevant to the muscle full signal if the restriction of myofibre expansion by the plasma membrane resulted in myofibre shrinkage and it is not immediately obvious why this would occur.

Whilst the phenomenon of the “bag-full “termination of protein deposition does occur, at least in terms of the response of MPS as shown in Figure 14, attempts to explain it in terms of signalling have by no means been resolved: in fact MPS falls whilst ic leucine concentration remains elevated and mTORC1 signalling is still active [338,339], at least those signals associated with the initiation of protein synthesis (p70S6K1, and 4EBP1). However, eEF2 phosphorylation does seem to increase coincident with the fall in MPS [339] and this indicates that the elongation phase of protein synthesis was inhibited, an effect observed in meal-fed rats in which the increased MPS in response to a meal was transient returning to baseline coincident with an increase in eEF2 phosphorylation [343]. Classically, eEF2 phosphorylation increases in response AMPK signalling associated with energy stress, such as muscle contraction, to protect ATP and PCr levels since it is the translational phase of MPS which is so energy dependent [308]. In meal-fed rats, post meal supplementation with either leucine or carbohydrate prevented eEF2 phosphorylation and extended the increase in MPS [295]. However, in human muscle the “bag-full” return to baseline of MPS occurs with no indication of inhibitory AMPK signalling or energy stress in terms of muscle ATP or phosphorylcreatine concentrations [339] so that the triggering of this response is unexplained. Nevertheless, it represents the only observed response which could be involved in mediating the inhibition of MPS.

Taken together, while the “bag-full” termination of postprandial protein deposition in muscle has clearly been shown to occur, its mechanism remains to be identified.

As for bag enlargement, there is little experimental evidence for the sequence of events during child growth, but as indicated above, (see legend to Figure 1), increased muscle collagen synthesis is observed as an early response to stretch-overload induced muscle growth in the fowl [61], and similar responses are observed in adult muscle stimulated by various intense exercise regimes which induce muscle growth as discussed elsewhere [269].

## 6. Nutritional Sensitivity of Growth of Muscle and Stature to Dietary Protein in Children

On the basis of the above discussion any consideration of the effect of dietary protein on muscle mass in childhood must be considered in terms of both indirect effects mediated through changes in bone-length (height) growth and associated muscle stretching, and direct effects on muscle to the extent that they can occur. The question of whether protein intakes influence length and muscle-growth in infants and children, as it does in animal models, and if it does, what is the optimal intake in infants and children, is in fact a complicated issue.

### 6.1. The Breastfed Child as the Normative Growth Model

Standard values for child growth published by WHO in 2006 explicitly identify breastfeeding as the biological nutritional norm and establish the breastfed child as the normative model for growth and development up to 5 y of age [344]. Although the values were derived from affluent children of non smoking mothers from widely differing ethnic backgrounds and cultural settings (Brazil, Ghana, India, Norway, Oman and the USA), linear growth was strikingly similar in the six sites [345]. Recommended protein intakes in infants and children are also anchored to the composition of breast milk and calculated for older infants and children with a factorial model based on maintenance and growth [346,347]. In fact, protein intakes are lower in breast milk than in most standard infant formulae and recommended protein intakes are considerably lower than observed intakes after weaning, i.e., ≤50% for most children in developed countries [348]. This raises the question of whether there are implications of these recommendations for height and muscle growth in children and following on from this, what is the lower limit of dietary protein intake necessary to mediate its anabolic drive on bone length growth?

### 6.2. The “Early Protein Hypothesis.”

The basis for the WHO breastfeeding directive (WHO, 2006; World Health Organization, 2016) was evidence of its benefit in terms of both immediate protection from morbidity and of longer term benefit for IQ in adolescence, and protection against childhood obesity and associated cardiovascular risk [349,350,351]. For example, in a most recent multinational 12-country cross-sectional study of obesity risk for children aged 9–11 years, the multivariable-adjusted analysis which included maternal BMI, indicated a lower risk of both general obesity (OR = 0.76) and high body fat (OR = 0.6) for exclusively breastfed compared with exclusively formula-fed children [352]. A key issue is that rapid weight-growth in infancy associated with formula feeding [348] appears to be detrimental to long-term health. Thus, when insulin or urinary C-peptide levels are reported in infants, higher protein intakes from formula or cow’s milk is associated with higher insulin and weight gain but not always with length gain [103,353].

Koletzko defined this risk in terms of an “Early Protein Hypothesis” [354] in which amino acids from an increased protein intake increase insulin and IGF-1 to mediate weight gain and adipogenic activity. The issue is complicated because there is considerable variation in growth in the first 2 years of life when infants often compensate for intrauterine restraint or enhancement of foetal growth and consequently exhibit significant “catch-up” or “catch-down” growth before following their genetic trajectory. There is also a positive association between genetic obesity susceptibility and postnatal gains in infant weight and length [355]. Thus, although it is clear that children who show catch-up growth in early infancy are at increased risk of obesity at five [356] and 8 years [357], with a meta-analysis of individual-level data on nearly 50 K subjects from 10 cohort studies showing that the growth rate weight velocity in the first year of life predicted the risk of childhood and adult obesity [358], some variation in growth can be independent of dietary feeding mode and early protein intake.

In any case, whilst the benefits of breastfeeding are universally accepted, the issue of breastfeeding and later obesity remains contentious. Adequately powered randomised trials of breastfeeding would not be ethical so that evidence mainly derives from cross-sectional or cohort studies, of which a very large number have been published. In addition to the recent international cross-sectional study indicated above [352], a 2016 systematic review of 40 previous systematic reviews [359] found a consistent association of breastfeeding with a modest reduction in the risk of later overweight and obesity in childhood and adulthood but stated that residual confounding cannot be excluded. Confounding has been suggested due to the relationship between social class and breastfeeding [360,361,362]. Furthermore, the protection by breastfeeding of risk of later obesity was not supported by the PROBIT trial, a very large multicentre RCT of breastfeeding promotion versus standard practice in Belarus [362,363]. However, it has been argued that PROBIT compared groups with a rather modest difference in duration of breastfeeding, and was underpowered to detect any effects on later overweight [364]. In response to this, the investigators in PROBIT argued that since their trial was able to detect the substantial and statistically significant beneficial effects of breastfeeding on gastrointestinal infection, atopic eczema, and growth in infancy, (breastfeeding promotion was associated with faster length growth), and on cognitive development at the age of 6.5 years, the absence of any observed effect on mean BMI or risk of obesity cannot be attributed to insufficient statistical power but rather to a lack of causal effect [365].

Nevertheless, a recent review of the impact of dietary protein intake in early life on weight growth and obesity concluded that the associations between high-protein intake during the first 2 years and later obesity were confirmed by recent studies [366]. Furthermore, utilising a large dataset comprising the combined data of 6708 children from 11 high-income countries from 4 cohort studies [367], three BMI growth trajectory patterns over the first 6 years of life were identified: Class 1, (5%), exhibiting persistent rapid growth, class 2, (40%), followed an early rapid growth pattern and class 3, (55%), followed the normative, WHO growth pattern. Mean BMIs at 20 years of age were 32.9, 25.9 and 22.4 kg/m^2^, respectively. Infants who were breastfed for <3 months compared with ≥3 months had a significantly greater risk to be in class 1 or 2 growth trajectories associated with increased obesity risk (i.e., fully adjusted ORs of 2.66 and 1.96, respectively). Taken together, these studies have been utilised to support the “Early Protein Hypothesis,” and the programming of subsequent adiposity, so that breast milk, as a low-protein infant food, affords protection whatever influence on length growth it may have [368]. This latter issue is discussed in more detail below.

As far as the potential mechanisms of the relationships between protein intake in early life and growth, many mechanistic studies have focused on IGF-1 as a potential link between dietary protein and growth. However, these studies need to be considered in the wider context of current understanding of the endocrine correlates of growth.

### 6.3. IGF-1 and Length/Height Growth in a Paediatric Context

**Figure 15:**
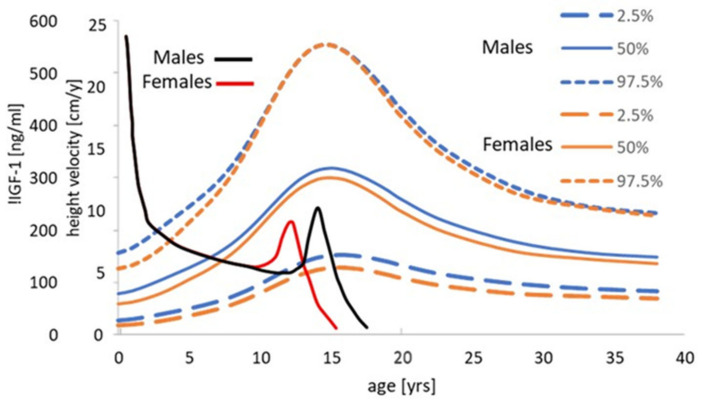
Serum IGF-1 and height velocity curves for males and females from birth to middle age [370]. It is clear that the overall pattern of changes in serum IGF-1 does not reflect height velocity apart from in puberty and even then, peak height velocity precedes peak IGF-1 by 2 years. Reference serum IGF1 percentiles for males and females calculated for a multicentre study with samples from 12 cohorts from the United States, Canada, and Europe (*n* = 15,014 subjects, 6697 males, 8317 females), with values obtained with an automated chemiluminescence IGF-I immunoassay. Values at birth from cord blood. Values drawn from Bidlingmaier et al. [370]. Height velocity curves for a cohort of Italian boys and girls [371].

There is an extensive body of literature with considerable differences between studies due to variation in assays. After a call for standardisation in 2011 [369], a standardised double monoclonal antibody was developed and used to generate reference values throughout the lifespan [370]. Values from birth to middle age in males and females are shown in Figure 15 together with a plot of height growth velocities through to the end of puberty in different children [371]. Within this large multicentre international cohort, IGF-I concentrations in cord blood samples from singleton pregnancies were significantly correlated to birth weight but not significantly different between males and females. In contrast, in a large population of Irish infants (*n* = 1000), cord-blood IGF-1 levels were slightly but significantly higher in females and adjusted IGF-1 Z-scores also correlated with weight (the strongest association, R^2^ = 0.19), as well as length, and head circumference [372]. After birth, IGF-I concentrations are lower than in cord blood during the first year of life, but after this values are higher in boys than girls, increasing gradually through the prepubertal period and more markedly afterwards to a pubertal peak, which occurred at 15 years in both boys and girls. However, when data were stratified according to Tanner stages, peak concentrations (50% percentile) tended to occur slightly earlier in girls, consistent with the earlier peak height velocity in girls apparent in Figure 15. Later in life, mean IGF-I concentrations were significantly higher in males than in females, although the difference is small.

What this means is that the overall pattern of changes in IGF-1 in childhood is unrelated to (or inversely related to) growth rates over the first decade of life, with the most rapid phase of postnatal growth coincident with the lowest levels of IGF-1, although concentrations do to some extent reflect growth velocity during puberty. However, peak height velocity shown in Figure 15 precedes peak IGF-I levels. In fact, another analysis of IGF-1 levels and growth in the same children showed that peak height velocity preceded peak IGF-I levels by almost 2 years, and, during puberty, multiple regression analysis indicated that serum IGF-I levels predicted height velocity in the following year [373]. Thus, the general relationship between IGF-1 and growth during the pubertal growth spurt is quite complex. Several, but not all, investigators report higher values in girls than boys [372,374,375] in infancy (in contrast to the reference values in Figure 15), but gender differences in IGF-I concentration are seen in studies mainly with older children (see Madsen et al. [375]). It has been suggested that gender dimorphism in IGF-I concentration developed between ages 3 and 12 months could reflect the gradual postnatal emergence of GH regulation of growth [374]. Finally, a quite complex gender difference in the IGF-1–growth relationship in relation to growth in leg length and trunk length has been described in school age children examined at 5 and 7–8 years of age in the Avon longitudinal study. Thus, although IGF-I was positively associated with total height and trunk length for boys and girls at both ages, in the older children the relationship with leg length was only significant in boys [376].

As for studies in which IGF-1–linear growth relationships have been assessed, a link is observed in some but not all studies and the link may be between IGF-1 concentrations and subsequent rather than coincident growth. IGF-1 levels in cord blood accounted for 19% and 7% of the variation in birth weight and length, and only 2% and 4%, respectively, at 2 months [372] while mean values from serial samples over 12 h in the first week of life accounted for 47% and 50% of the variation in birth weight and length, respectively [100]. However, in Indian newborn infants, cord IGF-I concentration was unrelated to length but reflected adiposity in terms of skin-folds at any birth weight as well as maternal milk intake at 38 weeks [377]. In the Cambridge Baby Growth longitudinal cohort study [374], with infants examined at 3 and 12 months, IGF-1 levels at 3 months were related to weight, BMI, and skinfold thickness but not length at 3 months and to all four measures at 12 months. Because higher IGF-1 levels at 3 months were associated with greater gains in length between 3 and 12 months and lesser gains in BMI and adiposity, the authors suggested a key role for IGF-I in the partitioning of overall infant weight gain into statural growth compared with adiposity. Recent studies in the Indian birth cohort of children support this view [378]. Thus, in contrast to their findings at birth (see above), at 2 years, IGF-I was positively associated with current length, but not BMI or adiposity. Additionally, in multivariate regression, 2 years IGF-I concentrations were positively associated with current length and milk intake. In older children in the ALSPAC prospective mother and child cohort study, IGF-I levels at 5 years of age predicted both gain in height between 5 and 8 years of age and insulin secretion at 8 years of age [357]. In this cohort, height at 8 years of age was positively related to BMI and insulin secretion which in turn reflected rapid weight gain between 1 and 8 years of age. Thus, although the authors suggested that IGF-I might regulate beta cell mass and insulin secretory response to glucose, given that insulin plays an important role in mediating hepatic GH-mediated IGF-1 secretion [84,98], the IGF-1–height relationship could in this case reflect a height–insulin secretion relationship rather than the reverse as suggested by the authors.

In other studies [379], while higher IGF-1 levels at 3 months predicted greater weight and height growth over the first 18 months, the associations were weak. In the recent EPOCH RCT [380], of low (LP) versus standard protein (SP) given from birth to 4 months, with a breastfed (BF) reference group, growth monitoring during the first 5 years of life was compared with postprandial insulin, C-peptide and IGF-1 concentrations reported at 0.5, 4 (primary outcome) and 9 months. The LP and BM groups were encouraged to use the LP formula as a follow-on formula from 4 to 12 months. Although IGF-1, insulin and C-peptide concentrations at 0.5, 4 months and 9 months were similar in SP and LP, (although higher in each case than in the BF group) growth in length was faster in SP compared with LP and BM group up to 12 months but similar in all groups after this (at 3, 4 and 5 years of age). The authors concluded that factors other than IGF-1 determined growth velocity at this age.

Michaelsen has reported IGF-1 levels and growth rates in several different cohorts of Danish children. In an observational study of healthy infants [381], IGF-1 at 9 months was related to weight but not length but was related to both at a 10-year follow up. In another Danish infant cross-sectional study of diet and growth at 2.5 years of age, IGF-1 did reflect height although explaining only 4.5% of the variance after control for weight and sex [382]. In an intervention study with whole milk or formula from 9 to 12 months of age [383], IGF-1 was not related to length at 9 or 12 months, but was related to weight at 12 months. In the SKOT cohort study of serum IGF-1, diet (7-day records), growth and body composition relationships were reported for data collected at 9 and 18 months [375] and subsequently at 9 and 36 months [384]. In the first report, IGF-I levels at 9 months, adjusted for gender, number of breastfeeding per day and weight at 9 months, were not related to length at 9 or 18 months but were related to the change in length from birth and from 9 to 18 months. In the second report, IGF-1 at 9 months and 3 years was related to height at 3 years.

One important feature of IGF-1 levels in infants which demonstrates that serum IGF-1 levels are not a simple predictor of either linear growth or protein intake is the differences observed between formula-fed or breastfed infants. All investigators report lower levels with breastfeeding [102,374,379,380,384], in some cases much less than half the values in standard formula [102] and lower than values in infants fed low protein formula [102,380] suggesting that the lower hormonal response of breast-compared with formula-fed infants is not just due to lower protein intake. The milk effect on IGF-1 levels is not a simple feature of the higher protein intake since an intervention with milk or a similar amount of protein from meat in prepubertal boys showed that only the milk increased fasting IGF-1 [104]. These authors [385] have argued that the milk effect on IGF-1 is mediated by casein, not whey, since their interventions with the casein and whey present in 1.5 L of skim milk in prepubertal boys showed an increase in IGF-1 only with the casein (given as an extra 40 g/day), and not with the whey (given as an extra 10.5 g/day). However, the actual increase in IGF-1 with casein was small (15%) and may well have simply reflected the marked differences in protein intake. They dismissed this by arguing that the results were not changed markedly after controlling for either protein intake or its marker serum urea nitrogen (which was 30% higher). However, the adjusted results were not shown and it is unlikely that such large differences in protein intake can be completely adjusted for statistically. Clearly more work is required to identify the nature of the milk effect on IGF-1.

Taken together, these and other studies indicate that the relationship between the endocrine effects of IGF-1, i.e., as clinically measured, and its para/autocrine influences at the growth plate are of relatively limited value in terms of understanding how longitudinal growth is controlled and any relationship with dietary protein intake. In reviewing paediatric implications of normal insulin–GH–IGF axis physiology, Bang [84], having stated, with little evidence, that changes in circulating endocrine IGF-I and paracrine/autocrine IGF-I are in most cases concordant, cites as an exception the example of the low serum IGF-1 in children with type 1 diabetes. In this case, peripheral insulin treatment results in a hepatic portal insulin deficiency, with reduced hepatic IGF-1 secretion and low serum levels. However, these children have normal linear growth as do mice with the liver-specific Igf1 gene knockout [113].

It is important to recognise that IGF-1 has multiple roles in energy metabolism and homeostasis, acting in an endocrine way on a wide range of tissues including adipose tissue [386,387] and in the context of the early protein hypothesis [354], the key action of IGF-1 was one with insulin, to increase adiposity. It is likely that this function is the most important in terms of the endocrine effects of IGF-1 as further discussed below.

### 6.4. Low Protein Intakes of the Breastfed Infant and Length Growth

The WHO length/height growth standards based on breastfeeding by affluent non smoking mothers indicate slightly higher growth rates than those of previous growth standards for both boys and girls (for a variety of reasons unrelated to protein intake [388]), and represent growth trajectories of healthy children achieving their genetic growth potential, and associated with optimal long-term health and development. Nevertheless, in the context of attempting to identify the relationship between protein intake, linear growth and consequent muscle growth, it is important to establish the extent to which linear growth can vary with protein intakes above that of breast milk (i.e., ≤6–7% protein calories).

In fact, in observational studies, length gain is lower among breastfed infants in some studies but not others [389]. A 2015 systematic review and meta-analysis of breastfeeding promotion interventions reported that although the effect of the interventions varied greatly among the studies, overall they induced a small, nonsignificant increase in length/height z scores, notably in children in the first six months of life [390]. In addition, the PROBIT trial of breastfeeding promotion did find slightly higher rates of length growth in the intervention group in early infancy followed by slightly slower rates up to 1 year of age but final heights at 16 months did not differ [362].

Importantly, there was no influence of protein intake on lengths/heights at 6 years in RCTs of LP versus HP formulas compared with a reference breastfed group [391]. In the EPOCH RCT discussed above [380], length for age z scores in the BM group were lower than for the LP group only at 4 months, and lower than the SP group from 4 to 12 months but were the same as both LP and SP groups after 1 year. Similarly, higher protein feeding of preterm LBW infants has not shown any sustained increase in length growth [392,393,394].

Prospective birth cohort studies of UK infants reported (a) exclusively breastfed infants at 3 months were smaller in weight, BMI and length at 12 months than those formula-fed in the Cambridge Baby Growth Study [374], and (b) breastfed children showed slower length gain than bottle-fed children up to 3 y of age but reached similar heights by 5 years in the ALSPAC cohort [395]. Additionally, some of the studies involving Danish infants reported by Michaelsen have indicated marginally lower length growth with lower protein intakes. Of 339 infants, all of whom were breastfed to some extent, length growth between 5 and 10 months was not related to total duration of breastfeeding or to protein intake but was slightly less in those who continued for ≥7 months [396]. Of 250 Danish healthy term infants, those still breastfeeding to some extent at 9 months consumed half the estimated protein intake of those who had ceased, and were shorter at 9 months, but only marginally so (*p* = 0.051), and were not different at 18 months [375]. Although this group reported no influence of whole cow’s milk compared with a lower protein formula on weight or length growth of 9–12-month-old infants [383], and no influence of protein as % calories on height in a cross-sectional study of 2.5-year-old children [382], in a cohort study of healthy infants to 9 months, with follow up at 10 years [381], length/height at 9 months and 10 years was related to protein intake (g/day and %E). However, in neither case was height related to serum urea nitrogen, which this group had previously shown to be related to protein intake [383], raising questions about the food intake measurements. Furthermore, after adjusting for infant body size at 9 months of age there was no effect of protein intake at 9 months on height at 10 years. In Dutch infants, protein intake at 1 year measured by a food frequency questionnaire was associated with greater height growth trajectories between 13 months and 9 years of age [397], although the effect was very small (a 25% increase in protein at 9 months was associated with a 0.2 cm greater height at 9 years). In a cohort of 1165 children in the Boston area of the US consuming a high protein diet (3.77 g·kg^–1^·d^–1^: ≥4 × EAR), in early childhood, (3.2 years), protein intake did not predict height in early adolescence although there was a trend towards a higher lean mass in boys [398]. Finally, a small study of Icelandic infants showed that any influences of protein intake at 12 months on length at 12 and 18 months ceased to be significant at 6 years [399].

Overall then it would appear that variation of protein intakes in infants or young children within the range of intakes provided by breast milk and infant formula or by current safe protein intakes have little if any impact on bone length growth. Thus, the “Early Protein Hypothesis,” idea of risk associated with high protein intakes and faster growth with protection by breast milk as a low protein infant food, seems to be true only in terms of the programming of excessive weight gain and adiposity, as proposed by Koletzko [368], and not for statural growth.

### 6.5. Height and Muscle Mass Growth of Human Children Is Limited to a Genotypic Maximum

Taken together, the above suggests that in infancy and early life, length growth is relatively insensitive to protein intakes at or above that of breastmilk at least in the moderate term, even though higher protein intakes may drive an anabolic increase in insulin and IGF-1, mediating greater weight (non-muscle fat-free mass and adipose-tissue) gain. It may be that the time course of longitudinal growth in infancy and childhood can be altered to some extent but the evidence would suggest that outcomes for height, at least by mid childhood, are not influenced by variation in previous protein intakes. This would indicate that, for the healthy infant of normal birth weight, breastfeeding followed by appropriate nutritionally adequate weaning foods, usually providing several times the RDA for protein over the first years of life, the observed length growth should be the genotypic biological norm for the child. As discussed below in relation to stunting, there is little to suggest that height growth of strictly vegetarian or vegan children in developed societies exhibit depressed height growth, even though their protein intakes are below that of most omnivore children. In terms of the Protein-Stat concept that muscle mass growth is determined and limited by bone length growth, if length/height growth in infants and young children cannot be increased to any great extent, then neither can muscle mass. This would explain why the excessive protein intakes of many formulas and complementary feeds often result in deposition of adipose tissue rather than muscle mass as suggested by the “Early Protein Hypothesis.”

### 6.6. Length/Height Growth and Dietary Protein in Older Children and Adolescents—The Issue of Milk Intakes

Identifying the specific role of protein in growth in childhood and adolescence is difficult because milk intake at these ages has been suggested to have a specific effect on height growth both in developing and developed countries, an influence not observed with any other animal source food. On the basis of a relatively limited number of cross-sectional, observational and intervention studies, Bogin reviewed the “Milk Hypothesis” [400] and concluded that increased milk intakes played an important role in the secular trend in human height. This idea was further examined by Hoppe, Mølgaard and Michaelsen [401] who concluded that milk has a height growth-stimulating effect even in situations where the protein and other nutrient intake is adequate. Such an influence on teenagers is supported by a large cohort study of US premenarchal girls followed for 6 years into adulthood. This showed that intakes of dairy milk, yogurt, and dairy protein were positively associated with height growth during the following year and that baseline intakes of milk and dairy protein were associated with PHV and with eventual adult height [402]. Recently, large-scale national cross-sectional studies of the association between milk intake and childhood growth in Chinese pre-school children [403] and school-age children aged 6–17 years [404] each reported that although milk intake was low overall, its intake was associated with increased height growth. Using a large sample (693 k) of children aged 6 to 59 months in low- and middle-income countries, milk consumption (yes or no in the previous 24 h) was associated with increased weight-for-age and height-for-age z-scores and a reduced prevalence of stunting, although the effect was small [405]. A recent study of major correlates of current male height in 105 countries [406] found that the human development index and total dairy protein intakes separately accounted for 71% and 64% of the variance.

It is the case that because of its specific biological function of enabling rapid postnatal growth, milk is a unique infancy food in having growth-promoting properties not found in meat or other animal source foods, although these properties are by no means understood. It is also the case that milk consumption by older human children and adults is, at least to a large extent, only allowed by lactase persistence which is the non-ancestral genotype [407]. Milk does have uniquely high levels of amino acids known to be involved in anabolic signalling. Tryptophan concentrations in the milk protein α-lactalbumin is uniquely high and the caseins and lactoglobulins contain high concentrations of leucine, (although this is not unique to milk with higher levels in maize and sorghum). In fact, on the basis of its high concentrations of tryptophan, leucine, glutamine, palmitate and microRNA-containing exosomes, Melnic [408,409] identifies milk as an evolved food signalling system that activates mTORC1 to mediate macromolecular synthesis, cell growth and development of the newborn mammal. One key issue is that although changing levels of consumption of dairy products in the 19th and 20th centuries may have contributed to secular national trends in height, with such increases associated with decreased overall mortality [2], there may be some negative pleiotropic consequences of milk consumption in terms of increased risk of some non- communicable chronic diseases such as some cancers [410] as a consequence of continuous hyperactivation of mTORC1 [409] and mediated by increased levels of IGF-1.

Thus, there are plausible arguments for the suggestion that cow’s milk and dairy products are a positive influence on growth in stature in children. However, as far as RCTs are concerned the evidence for this is not entirely obvious. Four systematic reviews have been published, two of them including a meta-analysis. De Beer [411] reported a systematic review and meta-analysis of controlled trials, most of which had serious limitations and were of low quality, which included children who were stunted, slightly stunted or with Z scores in the normal range, and found a small influence on height growth, with milk having more of an effect than other dairy products, and being more likely to be observed in stunted children and in teenagers. A 2017 systematic review of the effects of dairy consumption on body size, composition and bone properties in children and adolescents [412] examined 14 mainly good quality RCTs which focused on body size and composition, identified two reporting a significant effect on height and only one from 11 trials reporting a positive influence on LBM. A 2019 review which included a meta-analysis of RCTs of milk and milk-product intakes on growth and body composition of healthy children and adolescents aged 6–18 [413] identified 17 trials with 2844 children and adolescents and found that milk and milk-product interventions increased lean mass slightly (210 g *p* = 0.04), but did not influence height gain significantly. Finally, a 2019 critical and very extensive review of 94 cross-sectional, longitudinal cohort studies and RCTs of the role of milk and other dairy products in the development of obesity in children and adolescents [414] (finding very little evidence of a positive influence) examined 20 RCTs. However, because the focus was on obesity, the influence on height and LBM was only superficially examined and not mentioned for several listed RCTs in which these measures had actually been reported. Thus, height was mentioned only in seven trials one of which reported a positive effect, and with LBM mentioned in eight trials with only one positive effect. Taken together, it would appear that the findings of the 2019 meta-analysis [413] probably represent the most current and reliable analysis of RCTs of milk and dairy products demonstrating a small positive influence on the LBM but not on height. This is not to discount the observational and cohort studies discussed above which do indicate an influence on height, just that this influence has not yet been convincingly identified in RCTs.

### 6.7. Protein Deficiency and Stunting

As for length and muscle growth in young children at protein intakes below those of breastfed infants or below the RDA, i.e., in protein-deficient children, such intakes are rare in developed societies [49] and there is little evidence that vegetarian or vegan children consume protein intakes lower than the RDA [415,416,417,418,419]. One exception in developed societies are those communities which follow a macrobiotic diet, a non-supplemented, relatively restricted vegan diet. Retarded linear growth is observed, with faltering after 4 months to a rate which is 3.5 cm/year less than normal and which recovers if they are fed milk [420,421]. In developing societies, whilst reduced height growth (i.e., stunting) is widespread, it has proved very difficult to identify protein-deficiency-related stunting specifically, as opposed to environmentally related enteropathies and/or deficiencies of other nutrients important for growth such as zinc or iodine [120,422]. In fact, most mixed plant-based diets, especially those based on cereals, provide sufficient protein if consumed to meet the energy requirement as indicated by the studies on vegan children in developed societies identified above. Even the largely cereal-based diets reported for the rural, urban, slum and tribal communities in India, which exhibit high prevalence rates of stunting, appear near adequate in terms of protein and lysine intakes [423], with the stunting likely reflecting pre- and post-natal exposure to environmental pathogens. It is the case that diets based on nutritionally poor and low protein staples such as cassava and other starchy-root staples such as taro, sweet potato and Ethiopian ensete can result in protein intakes below requirement levels and are associated with increased prevalence of stunting [120]. In addition, the balance of foods in some mixed plant-based diets may be inadequate as far as linear growth is concerned since Chinese boys studied in the China Health and Nutrition Survey who consumed a plant-based diet (80% of protein from plant foods) grew taller when their diets provided a higher protein intake [424]. Finally, there is evidence that the protein quality of some cereal-based diets is inadequate since improving the protein quality of maize by selective breeding was shown some years ago to allow normal height growth in infants [425], and community-based trials in Ethiopia have indicated improved height growth [426]. Taken together, these data suggest that low protein or poor quality protein-based plant diets, which can occur, may not allow optimal height growth although in most cases zinc deficiency cannot be ruled out as an alternative or additional factor [427].

### 6.8. Can Muscle Growth in Children Occur Independently from Length Growth?

There are obvious sex differences during the adolescence growth spurt [30] which result in greater height-adjusted muscle mass in males compared with females, driven by the endocrine system as indicated above. In UK late adolescents, height adjusted appendicular muscle mass is 31% higher in boys than girls [20]. However, the question considered here is the extent to which dietary protein can drive an increase in muscle mass by “bag enlargement” independently from stretch-induced growth within the “muscle bag” model. There are several difficulties in answering this question.

Firstly, there is the problem of measurement of fat-free mass (FFM) as opposed to muscle mass. About half the FFM is non-muscle tissues and of these liver and other splanchnic tissue mass are known to reflect both food and especially protein intake [7]. Additionally, skin, another significant component of the FFM, increases obviously with weight gain and BMI. Thus, the report of increased FFM without a change in length growth in VLBW infants fed increased protein intakes (4.2 and 4.7 compared with 3.4 g/kg/day) [392] would certainly comprise mainly splanchnic organ mass rather than skeletal muscle which in any case is underdeveloped in infancy.

Secondly there is the problem posed by weight gain associated with positive energy balance as opposed to actual growth of the muscle mass. Some expansion of muscle mass occurs with overweight or obesity although the extent is uncertain. In obese adult men and women, FFM accounts for 40% and 27% of the weight gain, respectively, similar to the composition of the initial weight loss in response energy-restricting dietary regimes (40% and 34% FFM), responses similar to the composition of weight gain observed in overfeeding studies [428]. Since creatinine excretion increases linearly with increasing BMI in men and women [429], this implies that weight gain includes muscle. Although one long-term overfeeding study in men did show most of the LBM gain comprised skeletal muscle [430], for obese men [431], much of the increased FFM was associated with the truncal region with no detectable increase in arms or legs, consistent with no change in strength or work done during maximal exercise. Interestingly no FFM gain is observed in overfeeding studies on a low protein (6%) diet [432] supporting the idea that protein induced FFM gain is mainly splanchnic tissues.

Thirdly, there is a reverse causality problem. Thus, in the context of describing the regulation of muscle mass in a paediatric context in which dietary protein is viewed as “paramount for optimal muscle development” [3], one quoted report of an association between higher protein intake and higher FFM [433] actually reports that in obese 16-year-olds, the FFM and total muscle mass are the main predictors of both energy and protein intake and discusses this in terms of muscle mass as a determinant of appetite.

With these caveats in mind, the literature is mixed on increased protein intakes and FFM growth in older children. In a large prospective cohort study of Dutch children which aimed to extend the “Early Protein Hypothesis” with intakes in mid childhood [434], dietary protein intake at 8 years (on average 16.5% energy intakes) did not predict height at 10 years but did predict combined risk of overweight and obesity up to 10 years, weight which was mainly explained through higher FFM rather than FM. In contrast, in Danish 6-year-old children, dietary protein was unrelated to change in height or FFMindex (FFM/H^2^) over the subsequent 3 years [435]. A cohort study of German children [436] reported similar results in that a higher protein intake associated with meat intake at 10 years predicted a higher BMI and risk of being overweight at 10 years and 15 years in boys and girls: however, in this case poultry intake predicted increased FM while red meat intake predicted increased FFM. A prospective study of diet measured during puberty (9–10 years) and body composition in young adults (18–21 years) [437] assessed by skinfold thickness showed animal protein intake to be unrelated to either BMI, FMI or FFMI in boys but to BMI, %overweight, FMI and FFMI in girls although the differences were very small. A 16 weeks RCT of milk as a substitution for sugar-sweetened beverages conducted in overweight and obese pre-pubertal Chilean children increased protein intake by 13% and induced a small increased accretion of DXA-assessed lean, but not fat, mass and increased height in boys [438]. The extra FFM in the intervention group was only 1.2% of the baseline total and any muscle mass increase within this could have been height–growth induced. Longer term interventions in teenage girls with either dairy food [439] for 2 years in New Zealand, or milk supplementation for 18 months in the UK [440], which increased protein intakes by 32% and 20%, respectively, and increased BMD in each study, had no effect on height, weight, lean or fat mass. Taken together, none of these studies convincingly demonstrate dietary protein-induced muscle mass growth in excess of that associated with height growth.

## 7. Conclusions

**Figure 16:**
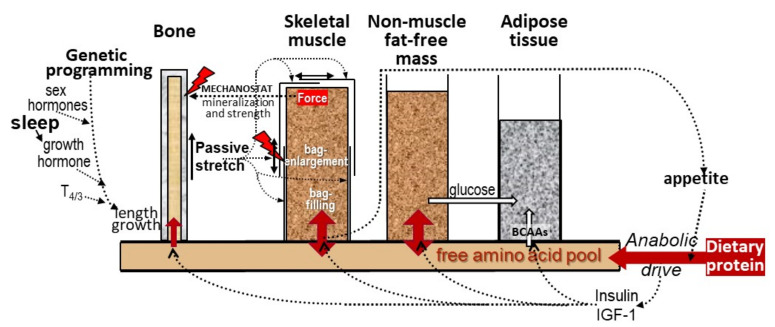
The Protein-Stat, (updated from Millward 1995 [7]). Whole-body protein content is controlled through an amino-static appetite mechanism, acting primarily to maintain skeletal-muscle mass at a level set by the linear dimensions of the organism. Bone lengthening occurs at rates determined by genetic programming (canalisation) mediated by growth hormone, secretion of which is primarily sleep-dependent, by thyroid hormones and by the sex steroids during puberty, acting together with an appropriate anabolic drive deriving from dietary protein. This anabolic drive, both permissive and active, is mediated by amino acids, insulin, IGF-1 (and in the rat T3) and other important nutrients like zinc and vitamin D, and provides the regulatory stimulus for protein deposition in all tissues. Net protein deposition during skeletal muscle growth occurs within myofibres which are limited in volume by the extracellular matrix of connective tissue which surrounds individual and groups of myofibres as well as the whole muscle (see Figure 3), like concentric “bags”. Thus, increased myofibre diameter and length during muscle growth requires remodelling of the muscle ECM to enable “bag” enlargement which is mediated by the passive stretching of skeletal muscle subsequent to bone length growth, activating satellite cells and other associated cells through mechanotransduction mechanisms. The linkage of bone length to muscle mass allows muscle size to be regulated at a genotypic muscle weight–bone length ratio. Increasing muscle size and force generation acts via the mechanostat to increase bone mineralisation and strength commensurate with muscle mass. Any dietary protein-derived amino acid intake in excess of that required for maximal “bag filling” will either expand the non-muscle lean body mass or be oxidized with the carbon skeletons leaving the liver as ketones and glucose, the latter to be taken up in adipose tissue for lipogenesis. Also excess branched chain amino acids can be taken up directly by adipose tissue and converted to fat. This metabolic fate of excess protein as adipogenesis is part of the Early Protein Hypothesis in which excess protein intake in infancy programmes adiposity.

This review has examined the details of the Protein-Stat model for growth control in which dietary protein drives appendicular bone length growth which drives muscle growth through stretch-induced mechanotransduction [7], from both phenomenological and mechanistic perspectives. The updated model, taking into account the mechanostat and other interactions discussed here, is shown in Figure 16.

As far as the first perspective is concerned, the most straightforward issue, namely the coordination of growth of muscle and stature, is clearly consistent with the model during puberty as demonstrated by the detailed longitudinal study of pubertal bone and muscle growth in Finish girls (see Table 1). Peak length velocity occurs first followed 1 year later by peak muscle cross-sectional area growth velocity, with mineralisation and consequent bone strength the last component of the bone–muscle growth relationship to occur. It is likely, but remains to be demonstrated, that this is the case in infancy and young children. However, the time-course of height and associated muscle growth at this stage of development remains uncertain, as are the extent of either cyclic changes in growth or the process of saltation and stasis, phenomena which have attracted little supporting or other evidence apart from the limited studies from the authors of works proposing them. While it might be suggested that the accelerated length growth during each saltation was necessary to achieve sufficient passive stretch for mechanotransduction of myofibre growth, this must remain speculative until saltation and stasis become a more widely accepted phenomenon and more information emerges on the linkage of muscle growth to length/height during such a process.

As to the nutritional sensitivity of growth of muscle and stature to dietary protein in children, evidence to date comprises a complex body of information, yet some aspects of the Protein-Stat model are clear. Overall skeletal muscle growth is linked to growth in stature with no clear evidence that it can be stimulated in excess of its expected genotypical height growth-associated trajectory by higher protein intakes or any other nutritional stimulus alone: i.e., stimulation of its growth by “bag enlargement” independently from stretch-induced growth has not been demonstrated. In adults, the linkage can obviously be broken by resistance exercise and there is no reason why this should not occur in children but this aspect of muscle growth regulation has not been examined here. While the FFM has been shown to increase in response to increased protein intakes any “excess” muscle growth, if it occurs, would be hidden as a minor component of the FFM expansion which does accompany weight gain. In fact, the inability to increase muscle mass in excess of its bone length-determined limit is an important concept in the context of the early protein hypothesis in which excess dietary protein intakes above that associated with normal statural growth is diverted to lipogenesis, programming subsequent adiposity. Clearly within the Protein-Stat model as originally elaborated, growth of the splanchnic organs and skin, which together account for more than half of the LBM, are responsive to dietary protein intake so that the limited evidence for an increased LBM not associated with obesity, with increased protein intakes, can be explained by an expected increased splanchnic mass. Increasing adiposity from protein intake in excess of the demand for muscle growth has been incorporated into the Protein-Stat model, (Figure 16).

The sensitivity of statural growth to dietary protein is not entirely resolved at least over the range of intakes at and above that provided by breast milk or the current RDA; protein intakes which are generally lower than those consumed by many children. In early life, any differences in length growth associated with feeding modalities appear to subsequently disappear in later years so that the pattern in time but not the overall extent may be responsive. The influence of milk intakes in childhood and adolescence remains as a unique stimulatory dietary factor, which some argue has mediated some of the secular trends in increases in human height. However, although some cohort studies have reported such influences, they have yet to be confirmed in RCTs. Clearly, protein intakes below that of breast milk or the RDA result in stunting in the relatively rare occasions when they occur but given the marked inhibitory influence of inflammation on the growth plate [120], which accounts for much of the stunting, the improvements in environmental safety and associated reduction in morbidity are likely to account for much of the secular trend in increase height.

As for the mechanistic perspective of the Protein-Stat model, when first proposed in 1995, stretch-induced muscle growth was well known but molecular and cellular models of mechanotransduction were only beginning to emerge. The work since then has mushroomed allowing much more detailed models of SC activation to be described and demonstrating a remarkable complexity both within and between the wide range of cells within the ECM of muscle. Thus, muscle growth is clearly amongst the most complex of any organ in the body. While SCs must be the major players in relation to providing the myofibre new myonuclei and an increase in its capacity for protein synthesis in early postnatal growth, this may be less important in later growth. Thus, during the extensive post-pubertal muscle growth observed in the male rat, when myofibre hypertrophy is occurring with an accompanying fall in protein turnover rate, a lessor increase in the capacity for protein synthesis is required for nuclear management of its expanding domain, with the main requirement being for ECM remodelling. This means that other cells amongst the mesenchymal stromal cell complement such as fibro-adipogenic progenitor cells can assume centre-stage. Whether this also occurs in later childhood and during the pubertal growth spurt remains to be discovered.

However, in contrast to the emergence of this detailed cellular and molecular biology of mechanotransduction, progress has been slow in evaluating other key elements of the model relating to the regulation of protein deposition in muscle at the level of protein synthesis. The central feature that muscle growth, in excess of that allowed in terms of “bag enlargement and filling”, is inhibited by the “bag-full” signal which overrides anabolic signalling by amino acids, is difficult to evaluate in animal models which exhibit continuous and rapid length growth. Clearly, the stability of the muscle mass in weight-stable adults (in the absence of resistance exercise) is obvious but it has taken difficult and innovative multiple muscle-biopsy, and stable-isotope studies of muscle protein synthesis in healthy adults during the fasting-feeding cycle to clearly demonstrate that the “bag-full” concept is real. However, such studies have yet to indicate the mechanisms involved.

As to the endocrinological regulation of bone length growth, many paediatric studies have focused on IGF-1 and its binding proteins as mediators of dietary protein influences, yet these studies remain difficult to interpret and problematic because of the uncertainties over the biological significance of circulating concentrations. Only in more recent years have the multiple endocrine roles of IGF-1 in energy metabolism and homeostasis become recognised, so that its significance as a mediator with insulin of increased adiposity in the context of the early protein hypothesis is probably the most likely interpretation of any relationship between serum IGF-1 levels and protein intake.

## Figures and Tables

**Table 1:** Timing relative to menarche (TRM) of peak growth velocities of lower leg muscle and bone during puberty in girls [43].

Parameter	Peak Growth Velocity	Growth State at 18 Years
	TRM (months)	
Tibial length (mm)	−20	complete
Total bone cross-sectional area (mm^2^)	−20	complete
Muscle cross-sectional area (mm^2^)	−8	incomplete
Cortical cross-sectional area (mm^2^)	−7	incomplete
Total bone mineral content (mg/mm)	−5	incomplete
Cortical volumetric bone mineral content (mg/cm^3^)	0	incomplete

**Table 2:** Size of the muscle myonuclear domain (protein/DNA) and protein synthesis *.

**A.** Male rat (gastrocnemius + quadriceps muscle) [255]								
Well fed								
Age	Protein	DNA	RNA/	Protein/	RNA × 10^3^		protein synthesis	
days	mg	mg	DNA	DNA	/protein	%day^−1^	g.day^−1^gRNA^−1^	g.day^−1^gDNA^−1^
23	49	0.35	2.1	141	14.8	28.6	19.2	40.3
46	145	0.87	1.9	167	11.3	16.1	14.2	27.2
65	230	0.9	2.1	255	8.3	11.5	13.8	29.4
130	453	1.12	2.1	402	5.2	5.3	10.1	21.2
330	1074	1.45	3.1	740	4.2	4.9	11.5	35.7
Marginally malnourished								
30	20	0.25	1	80	12.1	13.8	11.4	11.4
60	73	0.26	2.9	282	9.9	10.9	11	30.7
120	325	0.85	2.1	384	5.5	6.5	11.8	25
185	470	1.07	2.1	438	4.4	6.3	14.4	27.6
330	570	0.96	2.7	595	4.6	4.2	9.1	25
**B.** Adult Fowl (Gallus domesticus) ** [257]								
Posterior latissimus dorsi								
	240	0.26	3.05	909	3.4	6.9	20.4	62.1
Anterior latissimus dorsi								
	124	0.32	2.7	384	7.6	17	22.4	60.5
**C.** Changes, (% initial value), during load-induced hypertrophy of ALD [59]								
Days	Protein	DNA	RNA/	Protein/	RNA × 10^3^		protein synthesis	
	mg	mg	DNA	DNA	/protein	%day^−1^	g.day^−1^gRNA^−1^	g.day^−1^gDNA^−1^
1	10	25	11	−20	27	103	60	43
3	38	90	92	−23	153	122	−14	58
7	80	150	63	−31	125	71	−24	26
28	113	90	56	7.6	27	47	13	67
58	128	130	65	16	11	nsd ***	nsd	nsd

* Note that the values for protein synthesis, RNA and DNA are for the whole muscle and are average values for all cell types in muscle although the values will be largely representative of myofibre proteins and myonuclei. ** Rates of protein synthesis are likely to be higher in most avian species because of their higher body temperature: (41.7 °C for the fowl compared with 37.3 °C in the rat). *** Not significantly different changes from initial values.

## Data Availability

No new data were created or analysed in this study. Data sharing is not applicable to this article.
